# Response coupling with an auxiliary neural signal for enhancing brain signal detection

**DOI:** 10.1038/s41598-025-87414-9

**Published:** 2025-02-20

**Authors:** Ekansh Gupta, Raghupathy Sivakumar

**Affiliations:** Department of Electrical and Computer Engineering, Georgia Institute of Technology, Atlanta, GA 30332 USA

**Keywords:** Brain-machine interface, Electrical and electronic engineering

## Abstract

Brain-computer interfaces (BCIs) offer an implicit, non-linguistic communication channel between users and machines. Despite their potential, BCIs are far from becoming a mainstream communication modality like text and speech. While non-invasive BCIs, such as Electroencephalography, are favored for their ease of use, their broader adoption is limited by challenges related to signal noise, artifacts, and variability across users. In this paper, we propose a novel method called response coupling, aimed at enhancing brain signal detection and reliability by pairing a brain signal with an artificially induced auxiliary signal and leveraging their interaction. Specifically, we use error-related potentials (ErrPs) as the primary signal and steady-state visual evoked potentials (SSVEPs) as the auxiliary signal. SSVEPs, known for their phase-locked responses to rhythmic stimuli, are selected because rhythmic neural activity plays a critical role in sensory and cognitive processes, with evidence suggesting that reinforcing these oscillations can improve neural performance. By exploring the interaction between these two signals, we demonstrate that response coupling significantly improves the detection accuracy of ErrPs, especially in the parietal and occipital regions. This method introduces a new paradigm for enhancing BCI performance, where the interaction between a primary and an auxiliary signal is harnessed to enhance the detection performance. Additionally, the phase-locking properties of SSVEPs allow for unsupervised rejection of suboptimal data, further increasing BCI reliability.

## Introduction

Brain-computer interfaces (BCIs) enable direct communication between the brain and external devices, bypassing traditional human-computer interaction methods that require physical input. BCIs are either invasive or non-invasive: invasive BCIs, involving implanted sensors, provide high-resolution signals, whereas non-invasive BCIs, like Electroencephalography (EEG), allow for external recording without surgical intervention, offering a user-friendly and portable solution that is widely favored in research. Non-invasive BCIs primarily utilize techniques like EEG (Electroencephalography), MEG (Magnetoencephalography), fMRI (Functional magnetic resonance imaging), and NIRS (Near Infra-Red Spectroscopy), where EEG is widely preferred due to its cost-effectiveness, portability, and ease of use^1,2^. EEG captures signals like Event-Related Potentials (ERPs), Evoked Potentials (EPs), and Motor Imagery (MI)^3^ signals originating from the cerebral cortex and providing insights into sensory and cognitive processes^4,5^. ERPs, which are time-locked signals in response to specific external sensory stimuli, such as P300 and Error-Related Potentials (ErrPs), support applications like thought-based spellers, robot control, and error correction^6–9^, enabling advances in BCIs for both sensory detection and higher-level cognitive processing.

Event-related potentials (ERPs) such as P300 and Error-related potentials (ErrPs) are key signals in BCIs, often used in decision-making and error detection applications. ErrPs are evoked in response to perceived errors^10^, while P300 signals are generated during decision-making processes, typically via the oddball paradigm^11^. P300 is typically detected with 60–85% single-trial accuracy^12–14^ across studies, while ErrPs range from 60 to 75%^15,16^. Due to its utility in training autonomous agents^17^, error correction in human-in-the-loop systems^9^ and reinforcement learning^8^, ErrP detection has received significant attention, using spatial filtering techniques like xDAWN^18^ and Riemannian Geometry^15^, as well as deep learning models such as ConvNets and EEGNet^16,19^.

While BCIs have expanded beyond medical contexts into areas like security, entertainment, robot control, and VR^20–23^, certain challenges limit their broader adoption for general-purpose user applications. Non-invasive BCIs suffer from noise and artifact interference from sources like muscle movements and eye blinks, making accurate signal detection outside controlled settings difficult, thereby reducing practicality^24^. Furthermore, BCI devices often require careful, precise setup, complicating spontaneous use^25^. Signals also exhibit high variance and non-stationarity across individuals and environments, adding to detection challenges^26,27^. The complexity of brain signals also restricts BCI applications to specific tasks (e.g., spellers, error detection, attention shifts, etc.) rather than general-purpose use, as understanding unconstrained thought from EEG remains limited^28^. Additionally, brain data collection is costly and cumbersome, resulting in relatively small datasets and limiting broad application development^29^. A detailed overview of these challenges can be found in literature review papers^24,25,30^. Research continues to address these barriers, focusing on enhancing signal detection, transfer learning, and usability through spatial filtering^18,31–33^, machine learning^15,34,35^, and deep learning advances^16,36–40^.

To enhance practicality, dry electrode-based headsets have been developed to replace cumbersome headsets requiring conductive gels^6,41–44^. To reduce extraneous signals and artifacts in EEG systems, numerous solutions eliminate artifacts from sources such as eye blinks^45–47^, external movements^48–51^, and intramuscular signals^52,53^. Efforts have also been made to demonstrate the potential of BCI and EEG for certain general-purpose applications such as neural signal-to-imagined speech detection^54^, brain-to-text communication via handwriting^55^, speech perception^56^, and image recognition using neural signals^38,39^.

In this paper, we contribute to this body of ongoing research and offer a novel perspective on enhancing the detection of brain signals and gauging the performance effects by modulating the response of the brain using an auxiliary signal. Specifically, we propose response coupling, a method that aims to improve the accuracy and reliability of brain signal detection, by pairing a brain signal with another auxiliary brain signal and leveraging their interaction. This interaction-driven approach could pave the way for BCIs that are both highly accurate and adaptable to diverse, everyday environments. We provide a more detailed explanation and our motivation behind response coupling in the following paragraphs.

### Response coupling

Our method of response coupling is based on a simple idea: *Can the detection accuracy of a brain signal be improved by leveraging its interaction with another brain signal*? This question is derived by the insights from response competition^57^, a neural phenomenon where the simultaneous evocation of competing responses modifies certain properties (like latency) of the evoked signals^58,59^. Response competition, studied in the context of choice-reaction tasks, has also been shown to increase activity in certain brain areas when competing stimuli or responses are present^60^. Our motivation for an interaction-based detection system is also supported by extensive evidence in the literature on enhancing specific brain activity by reinforcing it with rhythmic neural activity. Rhythmic neural activity plays a pivotal role in various cognitive processes such as sensory perception, attention, and memory^61,62^, and reinforcing these oscillations linked to these functions has been shown to enhance the respective brain activities^63–65^. In this paper, we focus on ErrP signals as our primary signal of interest. We select Steady-State Visual Evoked Potentials (SSVEPs) as the auxiliary signal for this study. SSVEPs are neural responses that arise when individuals are exposed to periodic changes in light intensity, such as a flickering stimulus at a constant frequency^66^, with the response being phase-locked to the stimulus^67^. The choice to use SSVEPs is rooted in the aforementioned neuroscientific research demonstrating how rhythmic brain oscillations play a key role in strengthening cognitive functions. There are different explanations for the underlying mechanism behind SSVEPs, which are seen either as the superposition of transient neural responses^68^, or as a resonance effect in brain circuits tuned to particular frequencies^69,70^. Thus, modulating the primary stimulus with flickering stimuli may preferentially activate specific neural circuits by aligning with their resonant frequencies^71^, or modulate brain functions sensitive to the flicker frequency^72^. Although neuroscientific literature on the neural interactions induced by the concurrent elicitation of ErrPs and SSVEPs is limited, similar interactions have been explored in the context of other event-related potentials (ERPs) such as P300, particularly within hybrid-BCI applications^73–75^. These studies have provided some insights into the nature of their interactions. For instance, Li et al.^76^ investigated the mutual effects of concurrently eliciting SSVEP and P300 signals and found that SSVEP stimuli increased the amplitude and delayed the latency of P300 signals. Similarly, Wang et al.^77^, in their hybrid BCI setup, observed that concurrent SSVEP stimulation did not interfere with P300 signals. They reported that SSVEP amplitude was enhanced in the presence of P300, while P300 amplitude remained comparable to that in a P300-only BCI setup. Understanding the neuroscientific mechanisms underlying the interaction between ErrP and SSVEP signals is a critical and open area of research. Investigating these interactions could provide deeper insights into the neural processes involved and contribute to advancements in hybrid BCI systems. Although we have not encountered studies that explicitly address this interaction, we believe that such research would be valuable for the neuroscience and BCI communities. A deeper exploration into this topic remains an important direction for future work. To the best of our knowledge, no prior studies have investigated the concurrent elicitation of SSVEPs and ErrPs with the aim of enhancing ErrP detection accuracy.

**Fig. 1:**
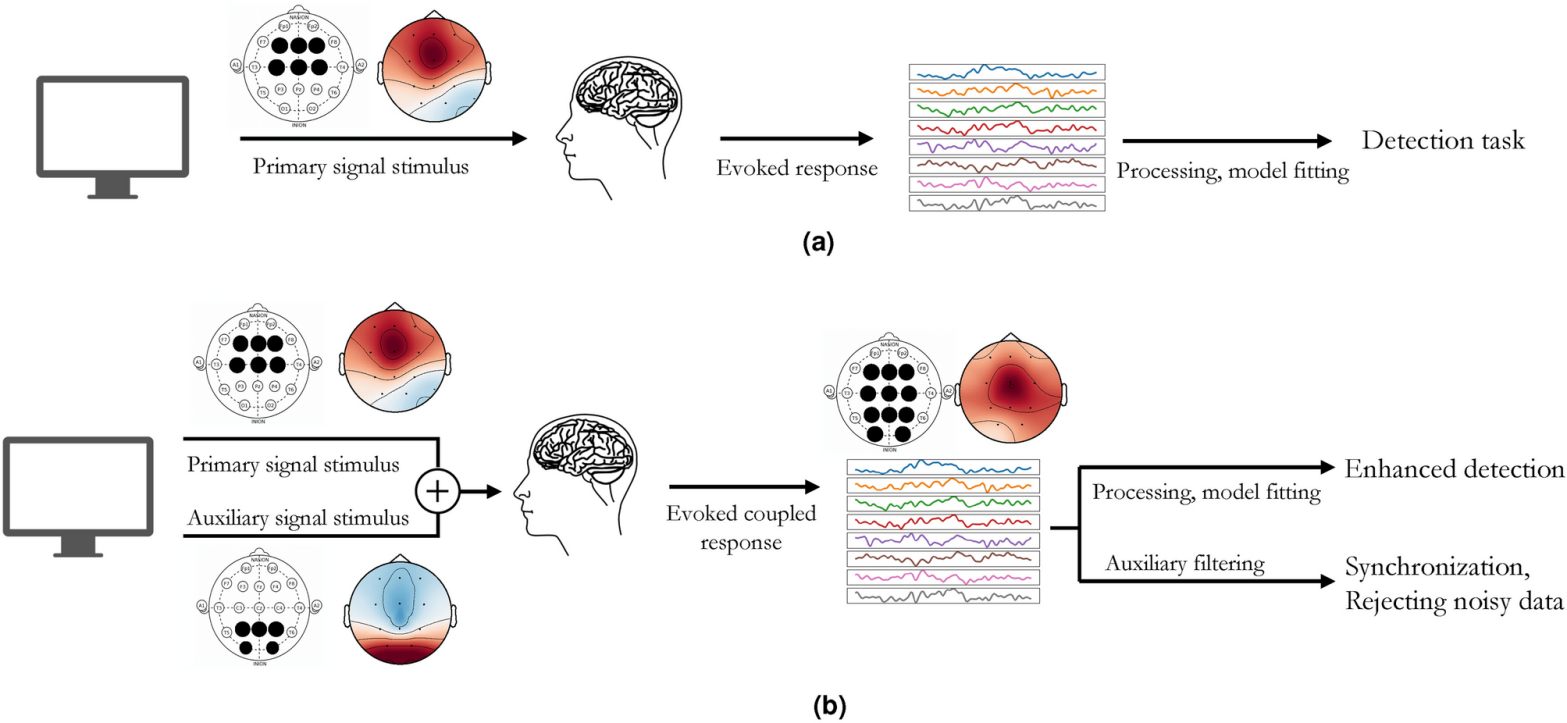
Response coupling schematic (**b**) in contrast with a typical BCI experimental schematic (**a**).

We refer to this method of combining signals as response coupling wherein the detection accuracy for a signal that is otherwise noisy and elusive is amplified by being considered in tandem with another signal (that is either artificially stimulated or is naturally occurring). This method differs from hybrid BCI systems, which use two or more signals in a redundant manner to independently compensate for each other’s shortcomings and improve performance^73–75,78–80^. A thorough overview of significant hybrid-BCI research can be found in some review studies^81,82^. A visual schematic of our method is presented in Fig. 1 where we contrast our method with a typical BCI experimental setup. We acknowledge that, while our method is likely applicable to other brain signal pairs, we entrust the validation of this assumption and a more in-depth exploration of the relationship between other brain signals to ongoing and future research efforts. In this paper, we demonstrate the results of response coupling ErrP signals with SSVEP signals by collecting two datasets in our lab on human subjects. Specifically, we demonstrate an improvement in the detection accuracy as well as the detection scope of ErrPs. While ErrPs are typically associated with activity in the Anterior Cingulate Cortex (ACC)^83^, pairing them with SSVEPs significantly enhances ErrP detection accuracy in the parietal and occipital electrodes compared to the baseline dataset. As a subsidiary advantage, we also demonstrate promising results in improving the overall reliability of the BCI experiment by utilizing the phase-locking properties of SSVEP signals to reject low-quality or noisy data in a completely unsupervised manner.

## Methods

### Datasets and experiment logistics

**Fig. 2:**
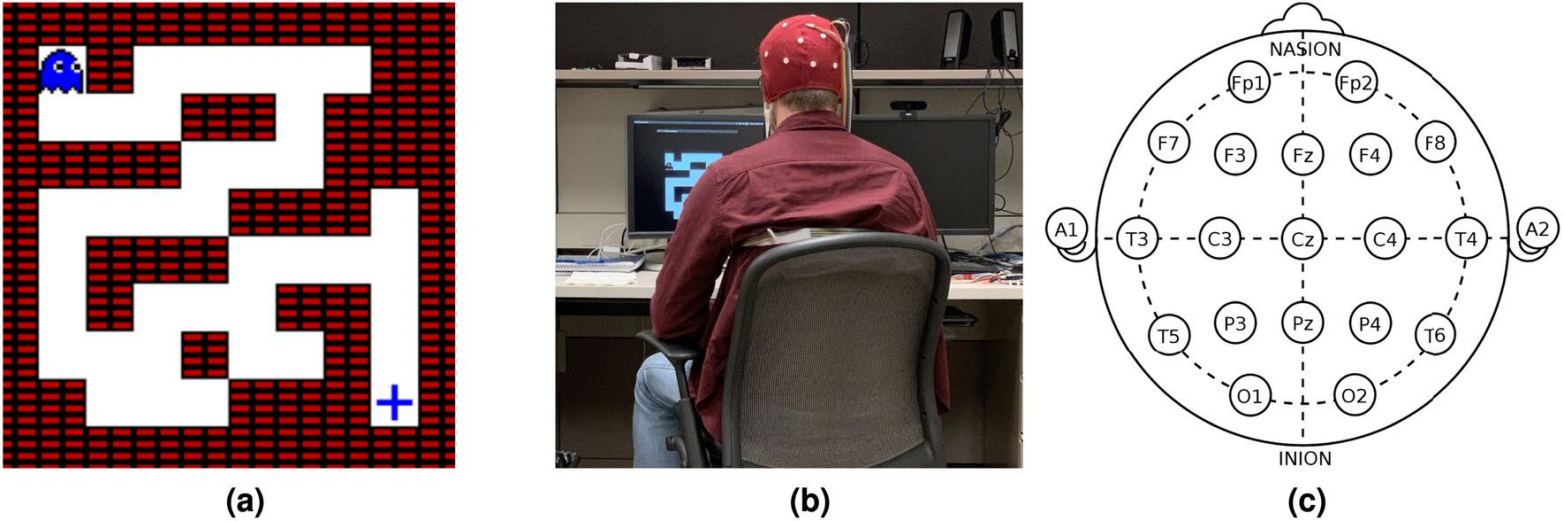
Experimental framework.

We collected two distinct datasets in our lab by using human subjects. To collect both these datasets, we employed a BIOPAC CAP-100C electrode cap, featuring 21 electrodes in the 10-20 electrode configuration distributed across the scalp (see Fig. 2c) to capture the electrical activity of subjects at a sampling rate of 125Hz. This cap was connected to the OpenBCI Cyton platform, which wirelessly transmitted data to a desktop computer. Due to the sensitivity of the electrode cap to even minuscule movements by the subjects and to ensure minimal interference from these inadvertent movements, participants were instructed to maintain a fixed gaze on the screen and avoid any motion other than breathing and blinking. They were seated comfortably to reduce movement as much as possible. Additionally, datasets were preprocessed to eliminate trials with movement-related artifacts. Specifically, trials were excluded if they exhibited a voltage spike exceeding 75$\mu V$ or a difference between minimum and maximum voltages greater than 125$\mu V$. This approach helped ensure that movement artifacts were minimized in the final analysis.

#### Experiment subjects

Across our two datasets (denoted by D1 and D2 respectively), we used the data of a total of 26 subjects with a mean age of 27.1 years (ranging from 24 to 31 years, five female) that took part in the experiments. 12 human subjects (mean age 26.7, 2 female) participated in the data collection for the first dataset while for the second dataset, we recruited 14 different (to avoid bias from those in the first study) human subjects (mean age 27.5, 3 female). A typical subject participating in the experiment is shown in Fig. 2b. All subjects had normal or corrected-to-normal vision. They showed no signs of neurological or psychiatric disorders and all gave written, informed consent. All subjects were explicitly informed that flicker stimulation might lead to seizures in epileptics and reported that neither they nor any members of their families had ever suffered from epilepsy.

The participants in this study were all current graduate students at the Georgia Institute of Technology who volunteered to take part in the experiments. They were compensated for their time and were informed of their right to withdraw from the study at any point without providing a reason. To ensure consistency between the experimental and control datasets, several key factors were controlled, including the duration of the experiment, the age range of participants, and an approximate gender balance within the group. However, certain subject-specific variables were not standardized. For example, prior exposure to the experimental protocol and familiarity with video games were not controlled. Similarly, while age range and gender ratio were approximately balanced, other factors, such as handedness, were not accounted for. Psychological factors-such as fatigue, mood, motivation, and distraction levels-were also not explicitly controlled. Additionally, individual cognitive baselines were not standardized beyond the shared characteristic that all participants were graduate students enrolled at the same university. All experiments conducted in this study complied with all relevant guidelines and regulations, and were performed according to a clinical trial protocol approved by the Georgia Institute of Technology Institutional Review Board (IRB) under protocol H23428.

#### Data collection and experiment stimuli

Each experiment session involved only one subject at a time. Subjects were seated comfortably in a chair positioned in front of the experimental setup. To minimize external disturbances, the experiment room was isolated, with the door kept closed, and only the subject and a single experimenter were present in the room at any given time. An electrode cap was carefully fitted onto the subject’s scalp, and a conductive gel was applied to each electrode to ensure proper signal transmission. Prior to starting the experiment, real-time electrode data was monitored to verify that all electrodes were transmitting EEG signals effectively. As an additional sanity check, subjects were instructed to blink three times, and the EEG data from the Fp1 and Fp2 electrodes was inspected for the characteristic eye-blink signature to confirm system reliability before commencing the experiment.

Our first dataset (we denote it by D1) focuses on eliciting ErrP signals in the user’s brain. ErrP signals are elicited when a human subject observes, interacts, or becomes mindful of an error committed by an external entity or by themselves^8–10,17,84^. For this experiment, we used an Atari-based maze game designed with a 10x10 grid containing obstacles as seen in Fig. 2a. An AI agent navigates this maze aiming to reach the target (the blue plus sign) and is capable of moving in four directions: top, right, bottom, and left. The agent makes a move every 1.5 seconds. With a probability of 0.2, the agent makes incorrect moves, which triggers the ErrP signal in the participants’ brains upon observation. Participants were thoroughly briefed on the agent’s actions and rules before the experiment commenced. Each user’s signal data was correlated with the agent’s position and actions to link the brain signals to specific game states. The maze game was developed using OpenAI Gym and displayed on a screen in front of the user, minimizing any extraneous audio/visual distractions. EEG data were collected using the OpenViBE software^85^, which ensured accurate timing with the agent’s movements via a TCP port. Each subject completed 10 episodes and each episode concluded with the AI agent successfully navigating the maze. There was a rest period of 1 minute between each episode. The duration of the experiment per subject was approximately 45 minutes. This dataset is publically available where it can be accessed^86^.

While our first dataset contains only ErrP signals and serves as our baseline dataset, we modify the experimental setting for the second dataset (denoted by D2) where we introduce an additional SSVEP stimulus to elicit simultaneous competing responses. As SSVEP signals are elicited by a subject observing rhythmic variations of light at a fixed frequency such as flickering^87^, ErrP and SSVEP stimuli were combined by adding a 12.5Hz flicker component to the existing maze-navigation game setup. We used the OpenAI Gym framework to make the screen flicker with a frequency of 12.5Hz. This frequency was chosen as it was close enough to the Individual Alpha Frequency (IAF) of users, enhancing SSVEP clarity^88^ and also lay in the mid-frequency range for optimally triggering a stronger SSVEP response^89^. The screen flickered with a frequency of 12.5Hz for 1.6 seconds (completing 20 cycles) and after that, there was a period of 200ms where the screen stopped flickering. This was done to create an anchor to have a ground truth for periods where the SSVEP stimulus (the screen flickering) was not active (more details in results). Thus, in this dataset, each action window was 1.8 seconds long. Each subject performed 10 episodes. The duration of the experiment per subject was also approximately 45 minutes, like the first dataset. This dataset is included with this manuscript and will be made publically available in the near future. Data preprocessing involved filtering signals with a 4th order Butterworth filter (1Hz to 40Hz range) and selecting 12 electrode channels comprising the occipital, parietal, central, and frontal regions (O1, O2, P3, P4, Pz, C3, C4, Cz, F3, F4, Fz, Fpz).

### Performance analysis and explainability

#### Detection models

To compare the performance differences between the baseline dataset and response-coupled dataset, we use four state-of-the-art detection models. As we use these models for comparing the performance difference between two datasets, we anticipate that the choice of a different model or the effect of ensemble learning will impact both datasets’ performance proportionally. We use these four models as they have been state-of-the-art for the detection of ERPs like P300^18^ and ErrP^16^, as well as other EEG signals like SSVEPs^90^ and fatigue, attention,^91^ etc. To evaluate all of these models, we use the balanced accuracy metric ((TPR + TNR)/2), where TPR = True Positive Rate or Sensitivity, TNR = True Negative Rate or Specificity. We use balanced accuracy as it is an excellent metric for unbalanced classes which eliminates biased models that excessively favor either the positive or the negative class. As the probability of the agent taking an incorrect action is 0.2, the number of Non-ErrP signals is expected to be roughly 4 times the number of ErrP signals, creating a class imbalance. The details of these four models are as follows:

- **xDAWN + ElasticNet**: The first model is a spatial filtering-based approach that uses xDAWN filtering to find optimum filters for the dataset, then uses Riemannian Geometry to classify them^15^. For the xDAWN-based classifier model, the data of each user was used for calculating the per-user test accuracy computed using 5-fold cross-validation. Each user underwent 4 repetitions of cross-validation to compute their respective per-user accuracy resulting in 20 runs per user. The resulting accuracy from all the users was averaged to yield the dataset’s average accuracy. This method was repeated 10 times to obtain the dataset’s average accuracy and standard deviation.
- **EEGNet**: The second model is a CNN framework based on deep learning, EEGNet^16^. For this model, the data from all users was combined into a single pool, and training and testing data was sampled from this pool without replacement. The training:testing:validation ratio was kept at 50:25:25. Using this split, we ran a sweep with different permutations of EEGNet hyperparameters (percentage dropout, the length of kernel, depth, and filter sizes) to find the optimal set of hyperparameters for each spatio-spectral permutation and dataset combination. For these optimum values of hyperparameters, the training and testing were repeated 10 times to obtain the average dataset accuracy and standard deviation values.
- **EEG-Deformer**: This is a CNN-transformer-based model that incorporates a Hierarchical Coarse-to-Fine Transformer (HCT) block that integrates a Fine-grained Temporal Learning (FTL) branch into Transformers, effectively discerning coarse-to-fine temporal patterns^91^. This model has achieved state-of-the-art performance in cognitive EEG signals such as attention, fatigue, and workload detection. Like EEGNet, the training:testing:validation ratio was kept at 50:25:25. Using this split, we ran this model with the following set of hyperparameters (number of temporal CNN kernels = 13, number of CNN kernels = 32, transformer depth = 4, attention heads = 16, dimension per attention head = 16, MLP heads = 16, dropout = 0.5) for each spatio-spectral permutation and dataset combination. For these optimum values of hyperparameters, the training and testing were repeated 10 times to obtain the average dataset accuracy and standard deviation values.
- **SSVEPNet**: This LSTM based deep-learning model achieved state of the art performance for detection of multi-class SSVEP signals with the added constraint of limited data availability and shorter window lengths (0.5s and 1.0s)^90^. While this model is designed to classify different classes of SSVEP signals, we use it to classify ErrP signals in our baseline as well as response coupling dataset, owing to its superior performance w.r.t. shorter window lengths. In addition, this model had the added advantage of not requiring hyperparameter tuning as it works solely with the number of EEG channels and window length as inputs. Like EEGNet and EEG-Deformer, the training:testing:validation ratio was kept at 50:25:25. Using this split, we ran this model for each spatio-spectral permutation and dataset combination. The training and testing were repeated 10 times to obtain the average dataset accuracy and standard deviation values.

#### Attribution and explainability

In order to interpret the performance of each dataset and meaningfully attribute it to the relevant factors, we also use two explainability methods based on the two classifiers we use. For the xDAWN-based classifier, we visualize the optimal filters corresponding to ErrP and non-ErrP signals that have been computed by the xDAWN algorithm for each dataset and contrast them to see their differences. For EEGNet explainability, we use two methods to attribute the output of EEGNet to its input features. We choose DeepLIFT (Deep Learning Important FeaTures)^92^ and e-LRP (Enhance layer-wise Relevance Propagation),^93^ two techniques that generate attribution maps by propagating an output backward through the neural net to identify the most relevant features of the input which informs the classifier. DeepLIFT decomposes the output prediction by backpropagating the contributions of all neurons in the network to the input features while e-LRP assigns relevance scores to each input feature by propagating the prediction back through the network layers. We chose these two frameworks for model explainability as out of all feature explainability frameworks, DeepLIFT and e-LRP have been shown to perform the best for EEG signals^94^, which influenced our choice of these frameworks.

#### Spatial-spectral segmentation

Since we are examining the effects of combining two signals that occupy different frequencies in the spectral domain as well as are evoked from different sources spatially, we decide to segment the accuracy analysis along the spectral and the spatial dimensions. While ErrP activity is largely confined to the theta band (4-8Hz)^95–98^ owing to its importance in cognitive control^99,100^. ErrPs are also primarily evoked in the Anterior cingulate cortex (ACC) region of the brain^83^, SSVEP signals are largely evoked from the primary visual cortex^101^. Evaluating the accuracy of our datasets along these spatio-spectral configurations can be beneficial for multiple reasons. Firstly, segmenting the data along spatial and spectral dimensions can serve as a proxy for the neural origins and frequency-specific characteristics of these signals. This division is grounded in prior research on the functional and anatomical differentiation of ErrP and SSVEP sources: the ACC for ErrPs^83^ and the primary visual cortex for SSVEPs^101^. By separating the parieto-occipital and fronto-central regions we aim to isolate the contributions of the ACC (responsible for ErrP) and the visual cortex (responsible for SSVEP), thus helping to evaluate whether these sources interfere or complement each other in our dataset. Secondly, the spectral configurations allow us to explore the distinct frequency bands critical for each signal. ErrPs predominantly occupy the theta band (4-8 Hz)^95^, while SSVEPs are strongest in narrowband responses tied to visual stimuli^97^. By incrementally narrowing the bandpass filters, we assess the impact of excluding specific frequency components, thus probing the system’s reliance on ErrP-dominated or SSVEP-dominated signals. Thirdly, segmenting our signals along the spatial dimension also provides us with potentially valuable insights into reducing the number of electrodes for portable BCI systems as it allows us to identify the specific brain regions that contribute most to the classification accuracy, providing insights into the critical electrodes necessary for signal detection. In order to dive deep into the performance of our datasets along the spectral as well as the spatial dimensions, we segment and analyze the performance of the two datasets along the following two levels of permutations:

1. We divide the number of electrodes into three configurations, each representing an area of the brain. These configurations comprise all electrodes (we denote this configuration by E1 and it includes the O1, O2, P3, P4, Pz, C3, C4, Cz, F3, F4, Fz, Fpz electrodes), only the fronto-central electrodes (denoted by E2 and including the C3, C4, Cz, F3, F4, Fz, Fpz electrodes), and only the parieto-occipital electrodes (denoted by E3 and including the O1, O2, P3, P4, Pz electrodes).
2. We also divide the signal profile of the raw EEG into three configurations based on its spectral content by applying different filtering levels to the signal. This spans a broad filtering configuration (we denote this configuration by F1 and it uses bandpass filtering from 1 to 40 Hz as this region contains mostly all the frequencies of interest), a narrower filtering configuration which eliminates the theta band and therefore a large component of the ErrP signals(denoted by F2 and using bandpass filtering from 10 to 40 Hz as this region contains predominantly the SSVEP and higher order frequencies), and an even narrower filtering configuration which only contains the immediate neighborhood of the SSVEP frequency (denoted by F3 and using bandpass filtering from 11 to 16 Hz)

Combining these dimensions, we get 9 different combinations for evaluating the performance of our two datasets.

## Results

Our main results are three-fold. We first detail our results on the enhanced detection accuracy for the response-coupled dataset compared to the baseline. To explain these results in detail, we use the attribution maps to show increased discrimination in more electrodes as well as other differences between the two datasets. Secondly, we verify the phase-locking effect of SSVEP signals and calculate the phase difference between stimulus and response. Finally, we talk about how our method can be useful in enhancing detection even further by filtering out users with noisy and suboptimal data using a completely unsupervised SSVEP-based metric.

### Response coupling enhances detection performance for ErrPs

**Fig. 3:**
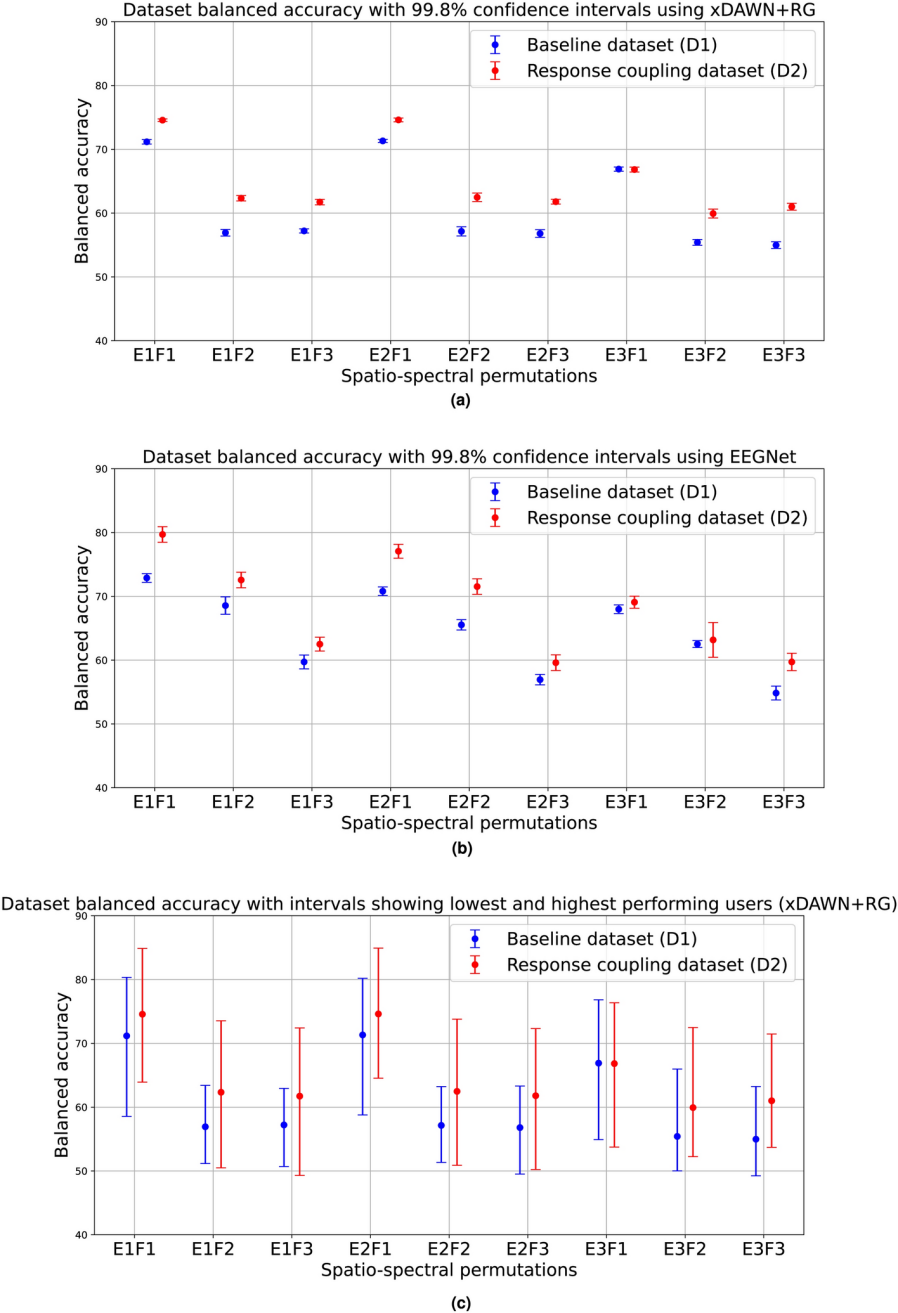
Dataset balanced accuracy for the spatio-spectral permutations using xDAWN+RG and EEGNet with confidence intervals and user accuracy ranges.

**Fig. 4:**
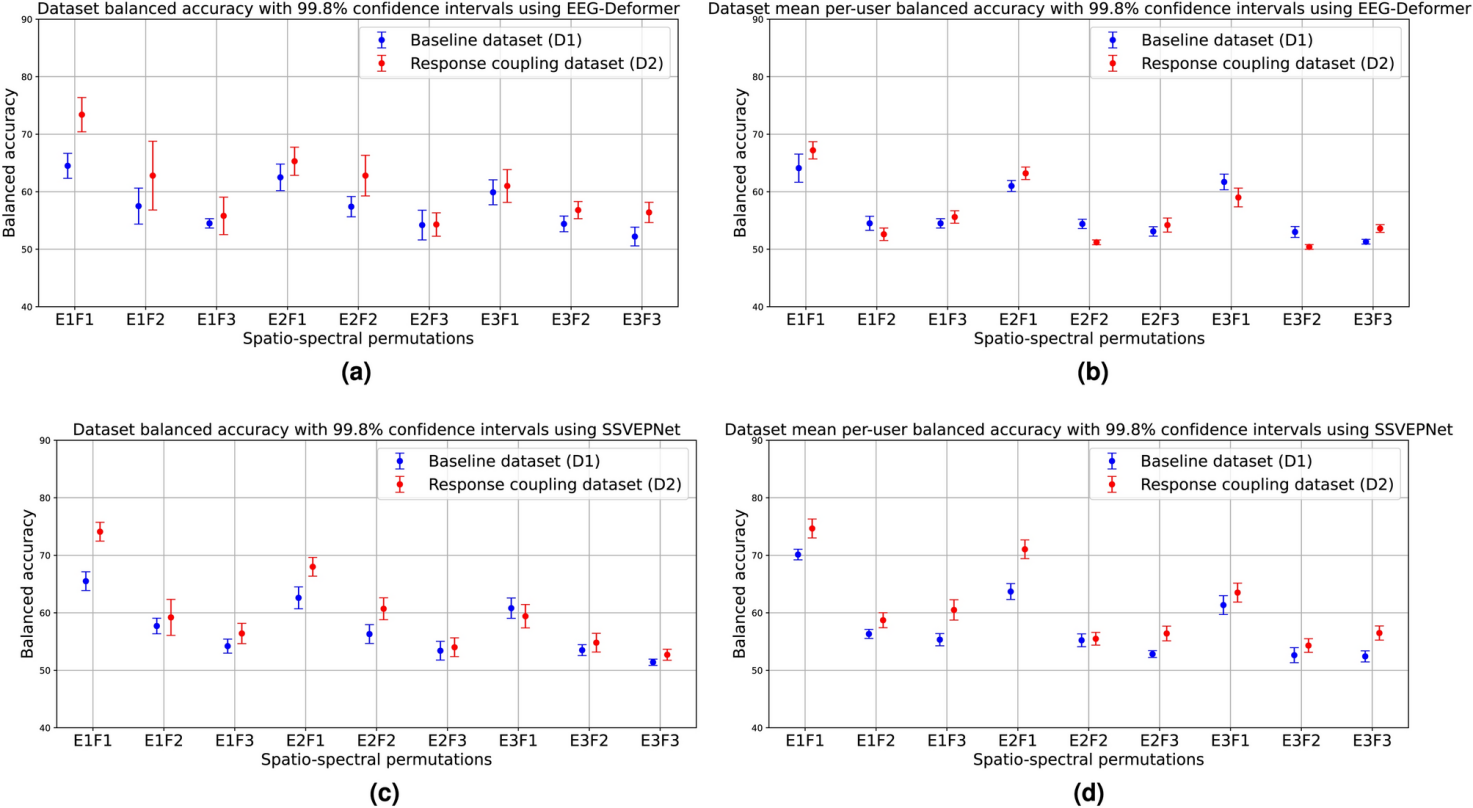
Dataset balanced accuracy for the spatio-spectral permutations using EEG-Deformer and SSVEPNet with confidence intervals.

**Fig. 5:**
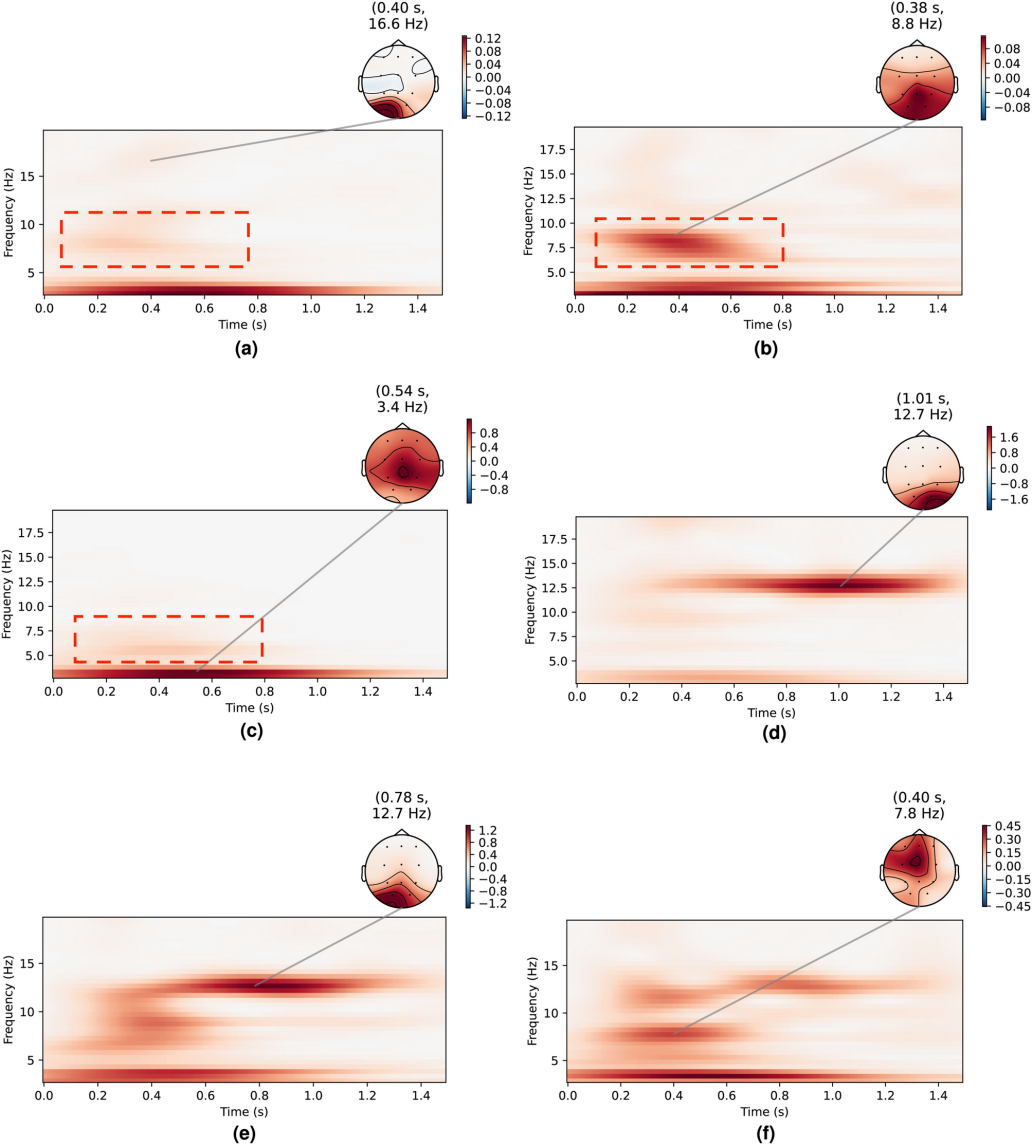
ErrP time-frequency plots for a few users in the baseline datasets (**a**–**c**) and in the response coupling dataset (**d**–**f**). For the baseline dataset, ErrP activity in the theta band (4–8 Hz) is highlighted.

We first present the accuracy levels observed for the 9 different spatial-spectral combinations in this evaluation. The detailed accuracies for these permutations are provided in Table 1. The table presents balanced accuracy values for the baseline dataset and the response coupling dataset. The values following the ± symbol indicate the standard deviation of balanced accuracy across multiple iterations of the aggregated dataset as described in the methods section. Figure 3a,b illustrate the dataset accuracies with 99.8% confidence intervals along the spatio-spectral permutations for xDAWN and EEGNet, respectively. Additionally, Fig. 3c displays the balanced accuracy values with intervals indicating the minimum and maximum balanced accuracies achieved per user in a dataset, representing the accuracy range from the lowest-performing to the highest-performing user in each dataset, using the xDAWN-based classifier. Fig. 4 also presents dataset accuracies evaluated using EEG-Deformer and SSVEPNet using two different testing configurations. Figure 4a,b demonstrate the mean balanced accuracy achieved when training and testing EEG-Deformer on the entire dataset vs when training and testing on individual users and averaging the per-user accuracies. Similarly Fig. 4b,c demonstrate the mean balanced accuracy achieved when training and testing SSVEPNet on the entire dataset vs when training and testing on individual users and averaging the per-user accuracies. We see that the response-coupled dataset shows higher accuracy and that it shows especially high accuracy compared to the baseline dataset for parieto-occipital electrodes for signals band passed between 11 and 16 Hz (thereby removing a large portion of ErrP activity) for three out of the four models tested. This suggests that the differential interaction of SSVEPs (originating in the posterior part of the brain) with ErrPs as opposed to the absence of ErrPs contributed to discrimination between ErrP and the baseline non-errp activity. This has fundamental consequences as we are able to detect ErrP signals farther away from the Anterior Cingulate Cortex (ACC), where it is evoked (Fig. 5)^83^.

**Table 1:** Mean balanced accuracy performance (in %) for response coupling ErrP dataset (D2) vs baseline ErrP dataset (D1). Each dataset is evaluated along 9 permutations of electrodes (E1, E2, E3) and band-pass filtering (F1, F2, F3) configurations.

Dataset	Model	E1F1	E1F2	E1F3	E2F1	E2F2	E2F3	E3F1	E3F2	E3F3
D1	xDAWN + RG	71.17 ± 0.25	56.93 ± 0.38	57.21 ± 0.24	71.32 ± 0.18	57.14 ± 0.53	56.79 ± 0.44	**66.90 ± 0.24**	55.41 ± 0.33	54.98 ± 0.39
D2	xDAWN + RG	**74.57 ± 0.15**	**62.33 ± 0.31**	**61.72 ± 0.32**	**74.61 ± 0.22**	**62.47 ± 0.49**	**61.79 ± 0.27**	66.83 ± 0.28	**59.93 ± 0.51**	**61.00 ± 0.40**
D1	EEGNet	72.88 ± 0.51	68.55 ± 1.03	59.72 ± 0.80	70.80 ± 0.47	65.53 ± 0.56	56.94 ± 0.64	67.98 ± 0.46	62.52 ± 0.39	54.84 ± 0.82
D2	EEGNet	**79.70 ± 0.94**	**72.56 ± 0.91**	**62.50 ± 0.77**	**77.07 ± 0.82**	**71.53 ± 0.94**	**59.60 ± 0.88**	**69.08 ± 0.65**	**63.17 ± 2.01**	**59.72 ± 0.99**
D1	EEG-Deformer	64.11 ± 1.79	**54.50 ± 0.96**	54.50 ± 0.67	61.01 ± 0.77	**54.45 ± 0.64**	53.12 ± 0.66	**61.71 ± 1.02**	**53.06 ± 0.75**	51.34 ± 0.36
D2	EEG-Deformer	**67.24 ± 1.13**	52.62 ± 0.82	**55.65 ± 0.80**	**63.23 ± 0.82**	51.23 ± 0.34	**54.26 ± 0.94**	59.01 ± 1.27	50.44 ± 0.39	**53.65 ± 0.58**
D1	SSVEPNet	70.12 ± 0.67	56.32 ± 0.58	55.32 ± 0.78	63.69 ± 1.01	55.21 ± 0.82	52.82 ± 0.44	61.34 ± 1.20	52.62 ± 0.96	52.42 ± 0.71
D2	SSVEPNet	**74.66 ± 1.21**	**58.70 ± 0.96**	**60.49 ± 1.30**	**71.04 ± 1.20**	**55.48 ± 0.82**	**56.39 ± 0.94**	**63.51 ± 1.21**	**54.30 ± 0.87**	**56.48 ± 0.91**

Greater values are in bold.

#### Response coupling enhances ErrP detection in the parieto-occipital region

**Fig. 6:**
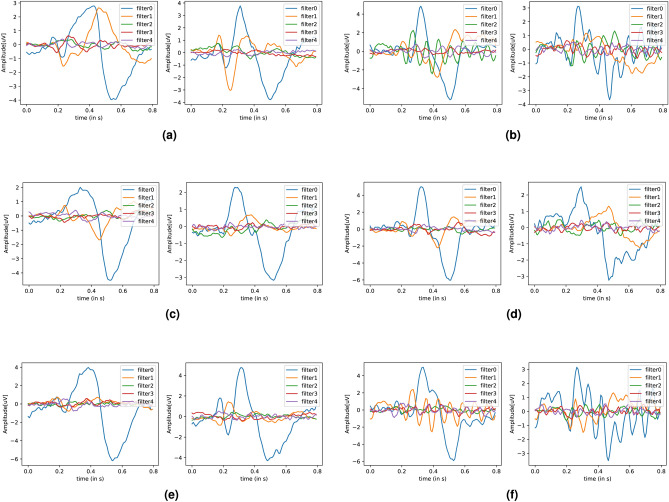
xDAWN templates for baseline and response coupling datasets for 1–40 Hz filtering and all, fronto-central, and parieto-occipital electrodes.

To explore this difference in greater detail, we visualize the class filters obtained from the xDAWN algorithm. The algorithm calculates the optimal class filters which capture the most salient features relevant to each class and which maximize the resultant signal SNR. These filters are visualized in Fig. 6. The figure is divided into 3 scenarios comprising all electrodes, only the fronto-central electrodes, and only the parieto-occipital electrodes respectively for both datasets. The signals in all these 3 scenarios are band-pass filtered from 1 to 40 Hz. As can be seen in the figure, the ErrP and non-ErrP templates show differential interaction with SSVEP signals (the rhythmic fluctuations observed for the non-ErrP template) for the second dataset, compared to the baseline dataset. This effect is non-existent in the baseline dataset, which is obvious, due to the lack of SSVEP signals. Additionally, this effect is seen to be maximally present in the parieto-occipital electrodes (elicitation region for SSVEP signals) compared to the fronto-central electrodes.

**Fig. 7:**
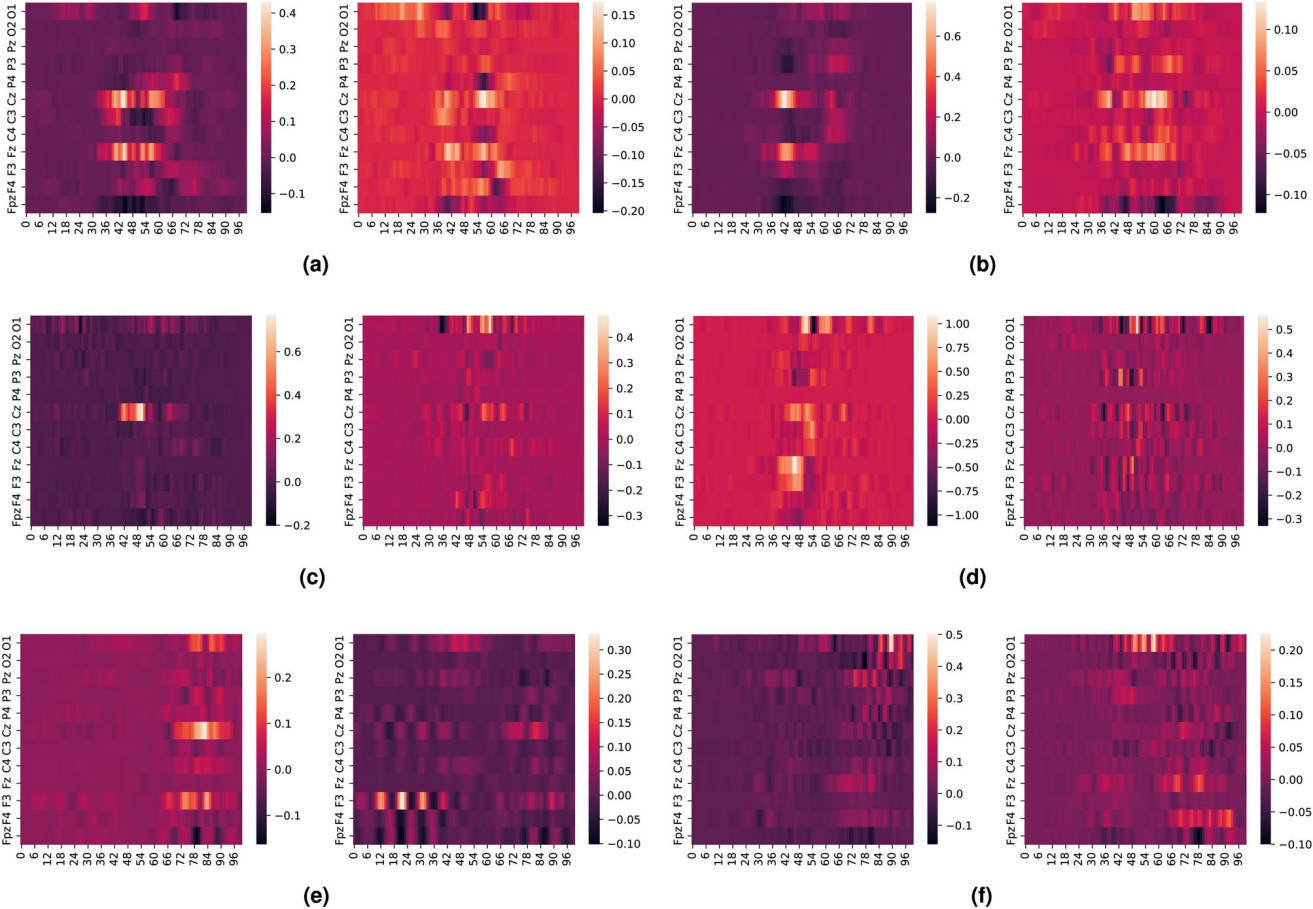
DeepLIFT activation maps for baseline and response coupling datasets for different levels of filtering and all electrodes. (**a**,**b**) show the activation maps for all electrodes bandpass filtered from 1–40Hz, (**c**,**d**) show the activation maps for all electrodes bandpass filtered from 10–40Hz, and (**e**,**f**) show the activation maps for all electrodes bandpass filtered from 11–16Hz. As seen in (**c**,**d**), ErrP activation maps show activations in the occipital electrodes for the second dataset which is absent in the first dataset. Additionally, the occipital electrode activations are larger for the second dataset than the first as seen in (**e**,**f**).
- For e-LRP, As seen in Fig. 8c–f, both the ErrP and the non-ErrP maps activation maps show larger activations in the occipital electrodes for the second dataset compared to the first dataset.

**Fig. 8:**
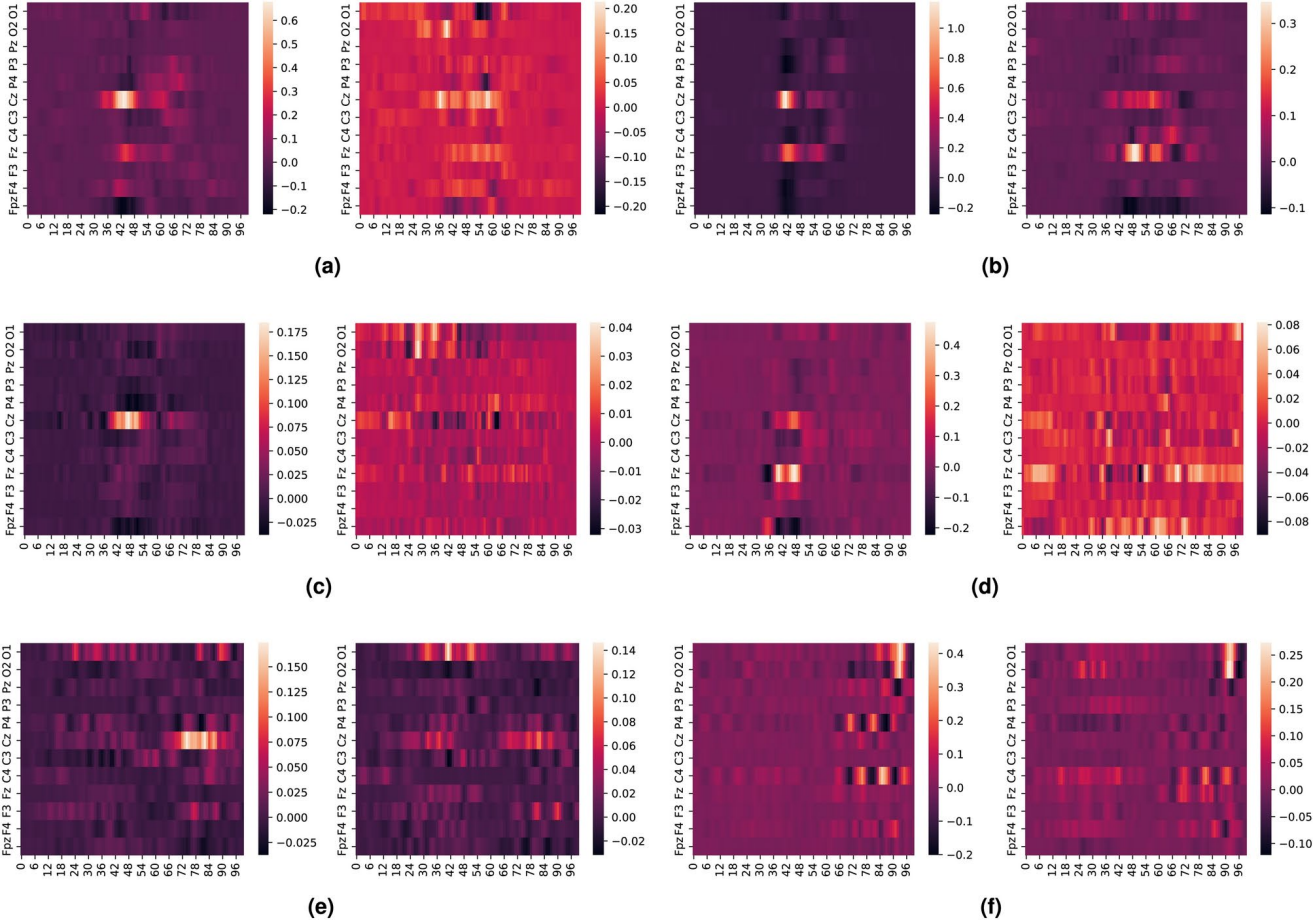
e-LRP activation maps for different levels of filtering and all electrodes.  (**a**,**b**) show the activation maps for all electrodes bandpass filtered from 1-40Hz, (**c**,**d**) show the activation maps for all electrodes bandpass filtered from 10-40Hz, and (**e**,**f**) show the activation maps for all electrodes bandpass filtered from 11-16Hz. As seen in (**c**,**d**,**e**,**f**), both ErrP and the non-ErrP maps activation maps show larger activations in the occipital electrodes for the response coupling dataset.
- For DeepLIFT and e-LRP, as seen in Fig. 9a–d, the activations for the occipital electrodes are much larger for the second dataset compared to the first dataset.

**Fig. 9:**
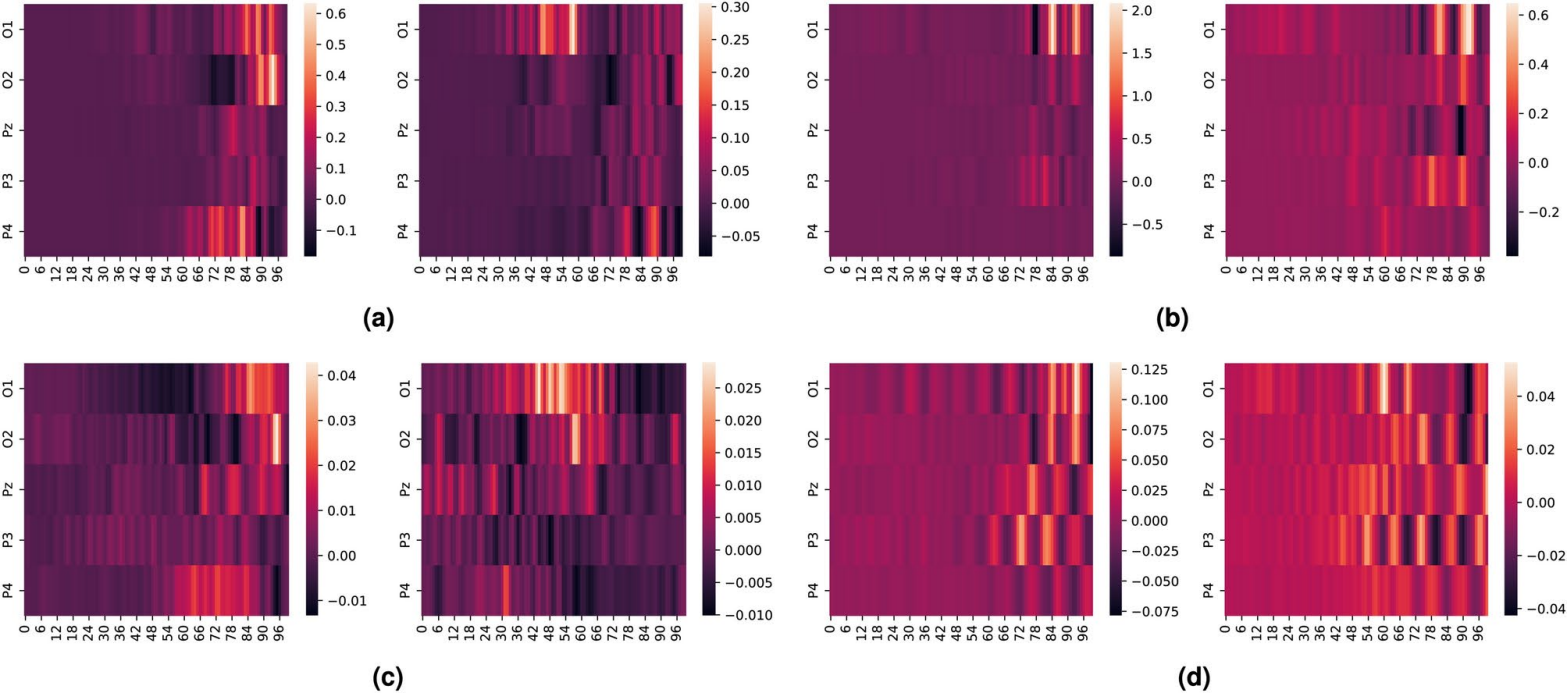
e-LRP and DeepLIFT activation maps for 11–16 Hz filtering for parieto-occipital electrodes. As seen in (**a**–**d**), the activations for the occipital electrodes are much larger for the response coupling dataset compared to the baseline.

We also explore the interaction between SSVEP and ErrP signals using attribution maps in EEGNet. We use e-LRP and DeepLIFT to create activation maps for the ErrP and non-ErrP samples which the EEGNet model correctly classified with the highest confidence (>90%). Since all the electrodes are visible in the generated activation maps, we generated these maps for the three filtering configurations to see their effect on the classifier as shown in Fig. 7 (DeepLIFT attribution maps) and Fig. 8 (e-LRP attribution maps). Also, to highlight the granular difference between EEGNet’s performance for the parieto-occipital electrode for the 11–16 Hz filtering, we also generated DeepLIFT and e-LRP activation maps for this scenario, as seen in Fig. 9

We notice the following characteristics in the attribution maps across different scenarios:

- For DeepLIFT, As seen in Fig. 7c,d, ErrP activation maps show activations in the occipital electrodes for the second dataset which is absent in the first dataset. Additionally, the occipital electrode activations are larger for the second dataset than the first as seen in Fig. 7e,f.

This suggests that response coupling not only facilitates enhanced ErrP detection in the parieto-occipital electrodes but also that the interaction between SSVEP and ErrP signals enhances the models’ ability to detect ErrP signals. This is particularly evident in the F3E3 scenario, where the response-coupled dataset enables the classification of ErrP versus non-ErrP signals solely based on SSVEP signals (those filtered between 11 and 16 Hz and recorded at parieto-occipital electrodes).

#### Generalization performance

ErrP signals exhibit substantial variability across tasks, environments, and sessions, posing significant challenges for model generalization across datasets, particularly in single-trial classification^102,103^. To investigate the impact of coupled signals on inter-subject classification, we compared the zero-shot classification performance of xDAWN and EEGNet models on both our baseline and response coupling datasets. Zero-shot classification accuracy in this context refers to the ability of a model trained on one user within a dataset to generalize and perform accurately when tested on a different user from the same dataset. To assess the generalization performance of our datasets, we evaluated two scenarios: (1) 1-v-1 generalization, where the model was trained on data from one subject and tested on another subject from the same dataset, and (2) 1-v-rest classification, where the training data for each user consisted of data from all remaining users in the same dataset. To ensure robust evaluation, all possible train-test subject permutations were considered in each generalization scenario. All subjects’ data was bandpass filtered from 1 to 40 Hz (F1 configuration) and all electrodes from the E1 configuration (O1, O2, P3, P4, Pz, C3, C4, Cz, F3, F4, Fz, and Fpz) were used for this analysis. Each evaluation was repeated 10 times, and the final results are reported as the mean balanced generalization accuracy with a 99.8% confidence interval. To ensure fairness, the same hyperparameters were used for EEGNet and xDAWN across both the datasets. The results, summarized in Table 2, indicate that the response coupling dataset demonstrates superior generalization accuracy, particularly in the 1-v-rest generalization scenario for both xDAWN and EEGNet models. In the 1-v-1 generalization scenario, the response coupling dataset also achieved higher accuracy with the xDAWN model, while the EEGNet model showed comparable performance across the two datasets.

**Table 2:** Mean balanced accuracy zero-shot generalization performance for D1 and D2 (in %).

Dataset	xDAWN 1 v 1	xDAWN 1 v rest	EEGNet 1 v 1	EEGNet 1 v rest
D1	57.94 ± 2.58	61.17 ± 1.54	**54.03 ± 2.76**	66.04 ± 1.45
D2	**62.09 ± 3.23**	**67.28 ± 1.32**	**54.28 ± 2.34**	**76.04 ± 2.54**

Greater values are in bold.

### SSVEP phase and rejecting noisy data unsupervised

In this subsection, we also use the SSVEP response to verify the phase-locking behavior of the stimulus as well as calculate the phase difference between stimulus and response. SSVEP signals are well known to exhibit a phase locking value (PLV) with the repetitive stimulus^104^. Phase locking has been used to boost the information transfer rate of SSVEP-based spellers^105^ by improving the number of targets by coding them w.r.t. frequency and phase^106^. While SSVEP signals mimic the stimulus frequency with a certain delay and phase lag, their elicitation is a result of the activation of several cortical networks^66^ due to the functional anatomy of the visual system, wherein, input to the primary visual cortex (V1) is distributed to higher areas (V2, V3, etc.)^107^. The phase lag associated with SSVEPs is primarily a function of the propagation delays introduced by these distributed sources^66^. Propagation delays above 30ms have been observed within the monkey visual cortex^108^ while the estimated delays in white-matter fibers are upwards of 50ms^109^.

**Fig. 10:**
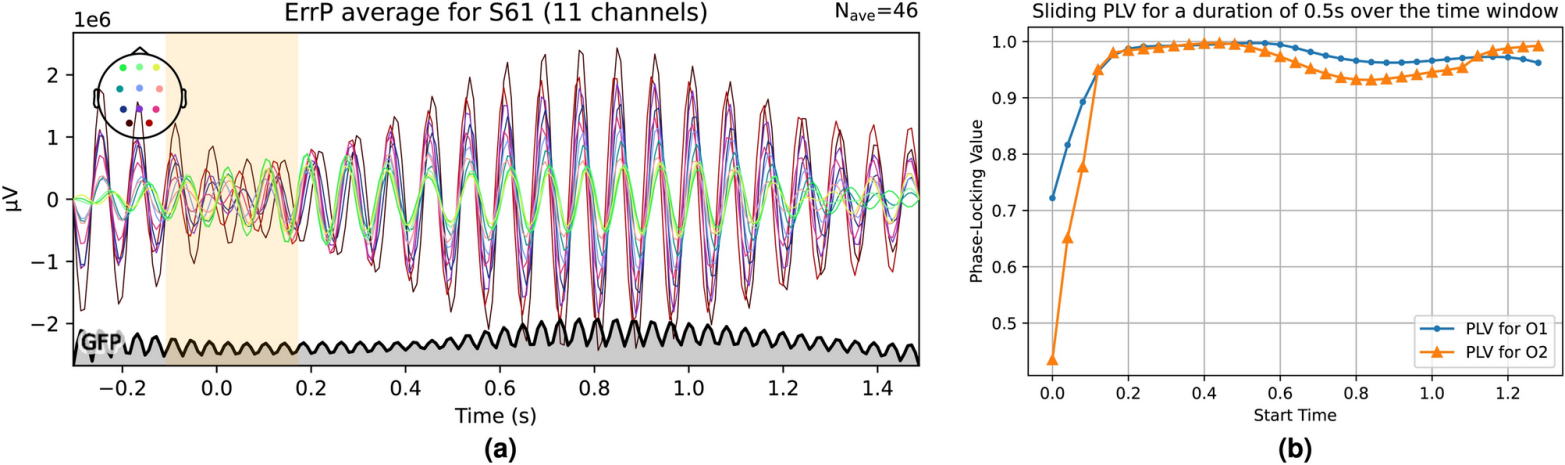
Average ErrP response signals bandpass filtered from 11 to 16 Hz for a typical subject with the 200ms non-SSVEP zone highlighted in yellow as well as the PLV as a function of time.

Based on this, we create a simple model for calculating the overall phase lag as observed in our experiments, based on a cross-correlation model. To this end, we first verify the existence of the no-SSVEP zone in the average signal as shown in Fig. 10. We denote this average response by $\bar{R(t)}$ and calculate the Phase-Locking Value for this template^110^ by taking the Hilbert transform of the signal as follows:

1 $$\begin{aligned} \bar{R_h(t)} = \frac{1}{\pi }\int _{\infty }^{-\infty }\bar{R(\tau )}*\frac{1}{(t-\tau )}d\tau \end{aligned}$$

The Hilbert transform is then used to create the analytical version of the signal as follows:

2 $$\begin{aligned} \bar{R_A(t)} = \bar{R(t)} + j*\bar{R_h(t)} = A_R(t)e^{j\Phi _R(t)} \end{aligned}$$

Let the active flickering component of the stimulus be denoted by the following:

3 $$\begin{aligned} S_0(t) = A_0*sin(2\pi F_0t), \quad t \in [0,T_0] \end{aligned}$$

We also create an analytical signal from the stimulus by taking its Hilbert transform and appending it as its imaginary part,

4 $$\begin{aligned} S_A(t) = S_0(t) + j*S_{0h}(t) = A_S(t)e^{j\Phi _S(t)}, \quad S_{0h}(t) = \frac{1}{\pi }\int _{\infty }^{-\infty }S_0(\tau )*\frac{1}{(t-\tau )}d\tau \end{aligned}$$

The instantaneous phase-lag is then given by:

5 $$\begin{aligned} \Delta \Phi _{SR}(t) = arg(e^{j\Phi _R(t)} - e^{j\Phi _S(t)}) \end{aligned}$$

And the aggregate PLV value for *N* samples is given by:

6 $$\begin{aligned} PLV = \frac{1}{N}\left| \Sigma _{t=T_0}^{T_1}e^{\Delta \Phi _{SR}(t)} \right| \end{aligned}$$

**Fig. 11:**
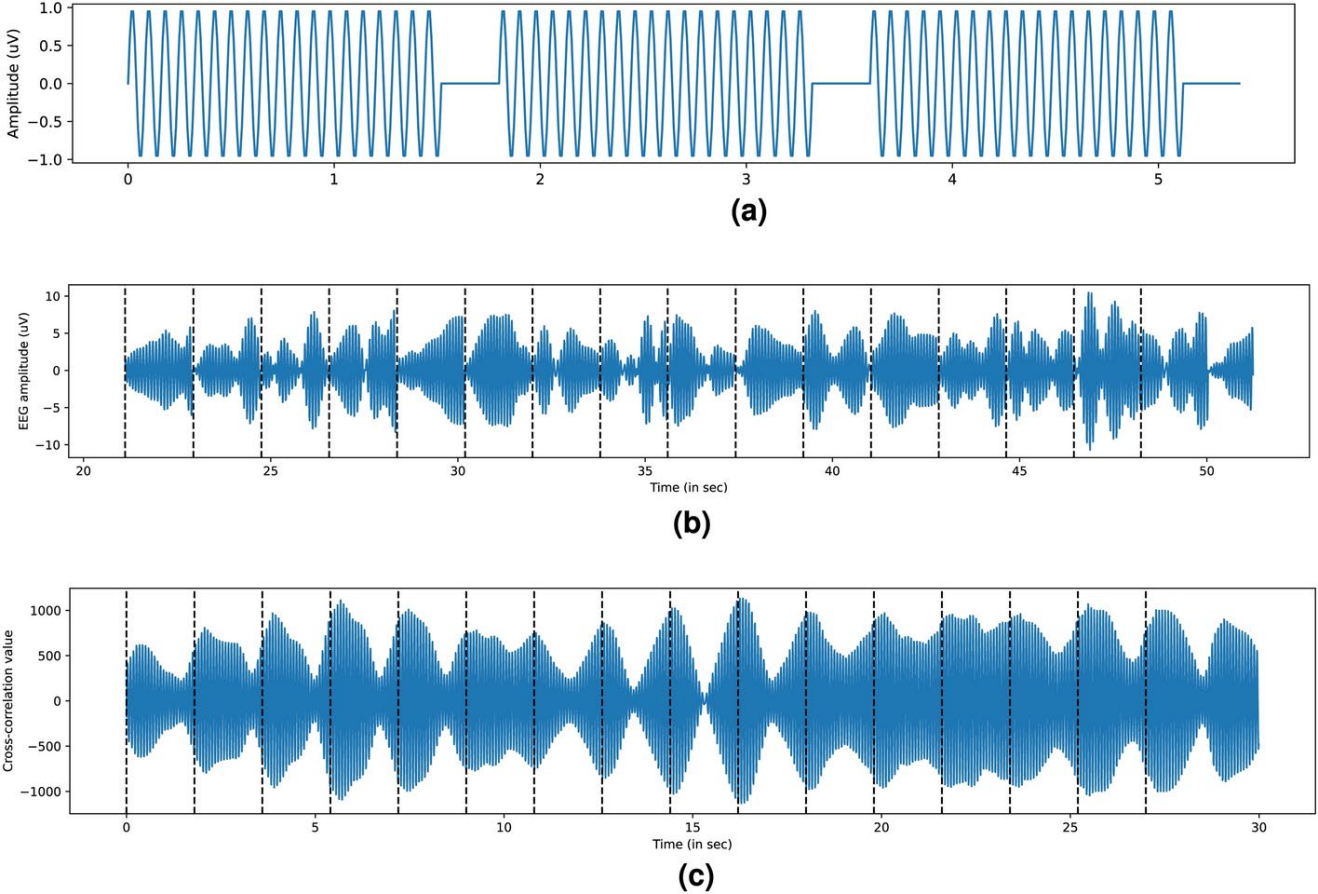
Stimulus signal cross-correlated with bandpass filtered O1 EEG.

An example of sliding PLV over 0.5 s (62 samples) within a larger 1.8-s window is shown in Fig. 10b. The initial 200 ms are ignored due to minimal SSVEP activity, while the high PLV indicates that the electrode’s response is consistently phase-locked with a near-constant phase lag. Our model calculates phase lag and response lag, as well as a fidelity metric, using cross-correlation between the response and stimulus. First, an analytical version of the stimulus signal (Fig. 11a) is created based on repetitions (1–150, with 3 shown in the figure). This signal is then cross-correlated with a band-passed (11–16 Hz) EEG segment from O1 and O2 electrodes. Within each action window (dashed lines in Fig. 11c), we identify the time delay corresponding to the cross-correlation maxima. These time delays reflect the phase lag, SSVEP frequency, and mean delay between the stimulus and response. A full derivation for this dependence is provided in supplementary materials attached to this article, which is omitted here in the interest of brevity. The final term includes $C(\tau )$, the cross-correlation at time-delay $\tau$; $F_0$, the SSVEP frequency; $\bar{\Phi }$, the average phase lag; $\bar{A}(t+\tau )$, the average EEG response (Fig. 10a); and constants $T_S$ time period containing active SSVEP stimulus (1.6s), *N*, and $A_0$.

7 $$\begin{aligned} C(\tau ) = N*A_0*cos(2\pi F_0 \tau + \bar{\Phi })*\left[ \int _{0}^{T_S} \bar{A}(t+\tau )*sin^2(2\pi F_0t)dt\right] \end{aligned}$$

This expression is maximized under two conditions: first, when $\tau$ aligns so that the high SSVEP amplitude within $\bar{A}(t+\tau )$ falls between $\tau$ and $T_S + \tau$, aligning the response delay to match the stimulus. Second, maximization occurs when the cosine term $cos(2\pi F_0 \tau + \bar{\Phi })$ is at its peak, which happens when the total phase is an even multiple of $\pi$.

8 $$\begin{aligned} & 2\pi F_0 \tau + \bar{\Phi } = 2n\pi , \quad n = 1,2,3,\ldots \end{aligned}$$

9 $$\begin{aligned} & \quad \tau = \frac{n}{F_0} - \frac{\bar{\Phi }}{2\pi F_0}, \quad n = 1,2,3,\ldots \end{aligned}$$

Denoting $\frac{\bar{\Phi }}{2\pi F_0}$ by $\delta$ and putting the value in for $F_0$, we see that this expression is maximized with a separation of 80ms:

10 $$\begin{aligned} \tau = (- \delta + 0.08n), \quad n = 1,2,3,\ldots \end{aligned}$$

**Fig. 12:**
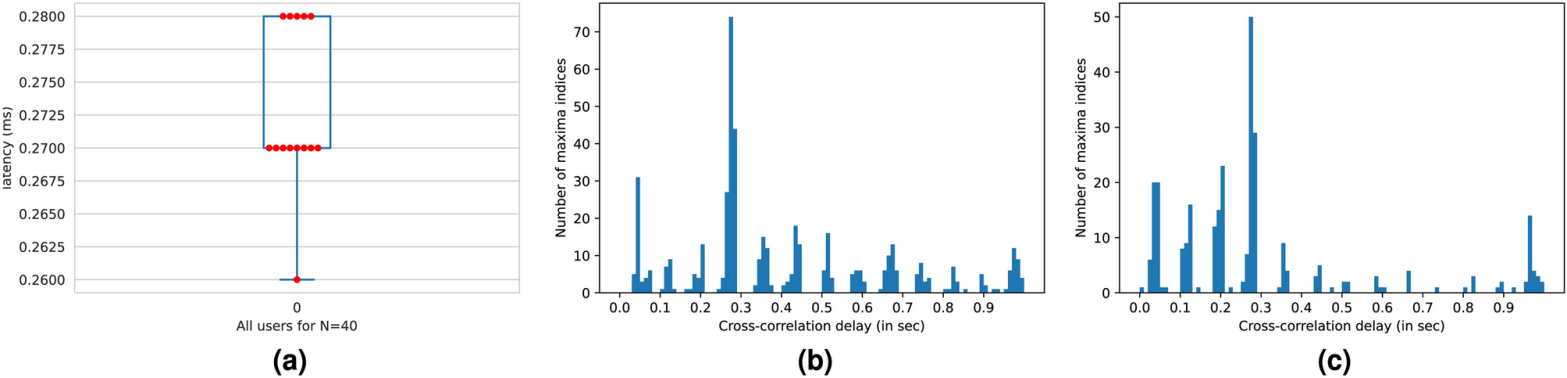
Most common delay zone boxplot and Histograms of indices of cross-correlation maxima for 2 users.

This behavior is seen empirically in Fig. 12b,c, where the maxima of the cross-correlation function appear at 80ms intervals. The most frequent peak delay across users clusters around 0.27s as shown in the boxplot for the most common delay zone for all users (see Fig. 12a). The synthetic signal simulating the stimulus is depicted in Fig. 11a, and a band-passed (11–16 Hz) SSVEP response from O1 is shown in Fig. 11b. This is cross-correlated with the synthetic stimulus to obtain a cross-correlation signal like in Fig. 11c. The offset at which this cross-correlation signal produces a maximum for each trial is then calculated and its histogram is seen in Fig. 12, where the maxima indices are quantized, consistently separated by 80ms-one period of the SSVEP signal, validating our equations. Experimentally, $\bar{\Phi }$ was approximately 4.05 radians.

### Response coupling detects impaired data completely unsupervised

The system developed herein is not limited to determining the phase lag and average delay between stimuli and responses but can also be employed to assess the level of impairment in EEG data. SSVEP signal responses are modulated according to the attentiveness of the subject^111^, with previous studies demonstrating their use in tracking attention^112^ and improving BCI performance^113^. In this context, we propose a novel, unsupervised metric termed the “fidelity score,” which estimates the quality of a subject’s signal data, as demonstrated empirically by subsequent results. This is achieved by contrasting the observed cross-correlation maxima latencies with those of a hypothetical ideal scenario, wherein the maxima indices of the subject’s data consistently occur at a fixed delay from the stimulus for every action window. The discrepancy between the expected and observed outcomes is utilized as an indicator to evaluate the level of noise and impairment in a specific user’s data during the experiment.

**Fig. 13:**
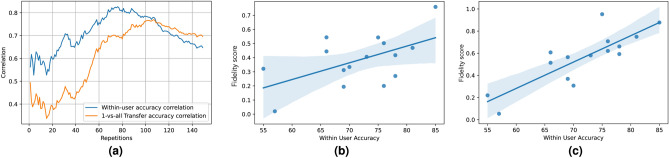
Variation of correlation score between accuracy and SSVEP-based fidelity score and visual representation of increasing correlation.

This metric is defined by estimating the most common delay zone, identified as 0.27s across subjects (see boxplot in Fig. 12a). Fidelity scores are computed for each user across various synthetic signal repetitions, from 1 to 150, and their correlation with both intra-user and one-vs-rest transfer learning detection rates is analyzed. For within-user accuracy, the correlation peaks at 0.83 (p< 0.003) at 100 repetitions, then gradually declines. Similarly, the correlation for one-vs-rest transfer accuracy peaks at 0.76 (p< 0.005) at 107 repetitions before declining. Figure 13a shows the variation in correlation scores as synthetic signal repetitions increase. Figure 13b,c depict the correlation scatter plot evolution from 15 to 75 repetitions, illustrating that fewer users fall outside the regression confidence intervals at 75 repetitions, with a stronger correlation between fidelity scores and data quality reflected by the steeper slope of the best-fit line.

## Discussion

We implemented response coupling to improve the detection accuracy, usability, and reliability in non-invasive BCIs by leveraging interactions between competing neural signals. Integrating response coupling with error-related (ErrP) and steady-state visually evoked potentials (SSVEP) enhanced signal discrimination in parietal and occipital regions, improving ErrP classification. Coupling ErrPs with SSVEPs also increased reliability and robustness by identifying users with poor-quality data without supervised testing. A proposed fidelity score based on SSVEP responses showed a strong correlation with data quality (0.83, p< 0.003), demonstrating its potential to ensure data quality and enhance usability.

This section outlines the rationale behind specific design choices, including parameter selections supported by experimental results. We selected an SSVEP frequency near the alpha band due to its association with attentional processes, as alpha rhythms support sensory processing by enhancing attentional focus^62,64,114,115^. For example, alpha power rises with cognitive load, reflecting distraction suppression during tasks like the Sternberg memory scan^116^. We also tested non-alpha frequencies to evaluate frequency selection effects by collecting data with varied frequency choices. Other parameters explored included the AI agent’s movement speed in the maze and the impact of different SSVEP flicker patterns on detection accuracy Our setup involved human subjects observing an AI agent navigating an Atari-based maze, with ErrP signals triggered by the agent’s errors and concurrent SSVEP stimuli at selected frequencies. This setup enabled us to evaluate the impact of different SSVEP frequencies and flicker patterns on response coupling, with results on accuracy and time-frequency analyses detailed in this section.

### Algorithmic design elements for response coupling

To develop an algorithmic framework aimed at enhancing the detection and characterization of brain signals, we outline the design elements and principles that guided our experiments. A key aspect of our approach involved devising a coupling task to facilitate interactions between primary and auxiliary signals. Below, we discuss the design dimensions and insights that informed our experimental setup:

- **Temporal independence**: We chose ErrP and SSVEP signals as our primary and auxiliary signals respectively as they can be elicited simultaneously and involve stimuli that can be overlayed on top of each other. In this context, two brain signals would be unfit for this experiment if the required perceptual and cognitive activity were mutually exclusive.
- **Spatial and spectral behavior**: We prioritized signals with differing spatial and spectral properties. This choice was motivated by the concern that if both signals were elicited from the same electrodes, it could complicate the separation of their components. Spectral independence, similarly, simplifies signal processing by making it easier to isolate the primary and auxiliary signals in the frequency domain. While this selection optimized signal separation, we acknowledge that further research could explore the interaction of brain signals with overlapping spatial or spectral characteristics.
- **SSVEP elicitation method and frequency**: Given the impact of the SSVEP frequency and elicitation method on the strength of the response, we experimented with additional flickering frequencies and techniques. In one variation, we employed screen flickering independent of the game’s action boundaries unlike our previous dataaset, so it was not synchronized with the AI agent’s movements. Additionally, we tested a peripheral flickering scheme using an LED strip around the screen edges, drawing from literature on radial flickering^117,118^. We also compared responses at 7 Hz and 14 Hz to evaluate the effect of low and another mid-range frequency^119^.
- **ErrP time window**: ErrP amplitudes are known to be stronger when participants experience less time pressure and prioritize accuracy over speed^120–124^. To explore this effect, we extended the action window from 1.5 to 3 s, reducing time pressure and giving participants more time to register an error mentally. This parameter allowed us to investigate how response coupling performs under reduced temporal constraints.

### Datasets with additional design elements

To investigate the effects of the ErrP time window, SSVEP frequency, and SSVEP elicitation method on detection accuracy, we experimentally collected three additional intermediate datasets that incorporated various combinations of these parameters. We denote the datasets as follows:

- D3—An ErrP-only dataset serving as a baseline, with a 3-second interval between successive AI agent actions.
- D4—This dataset added a screen-flickering component, which flickered independently of the maze game and was not time-locked to the agent’s actions the phase of the flickering at the agent action onset was independent of the game and thus was not fixed). The flicker frequency was set at 7 Hz to study the effects of using an SSVEP frequency in the theta band that overlaps with the ErrP spectral response^95–97^ and the action time was kept the same as D3 (3 seconds).
- D5—This dataset used an LED strip-based peripheral flickering component which illuminated the periphery of the screen instead of the screen itself. This flickering was also independent of the maze game and not time-locked to the AI agent’s actions. The flicker frequency was set at 14 Hz to explore the effect of choosing a mid-range SSVEP frequency

**Fig. 14:**
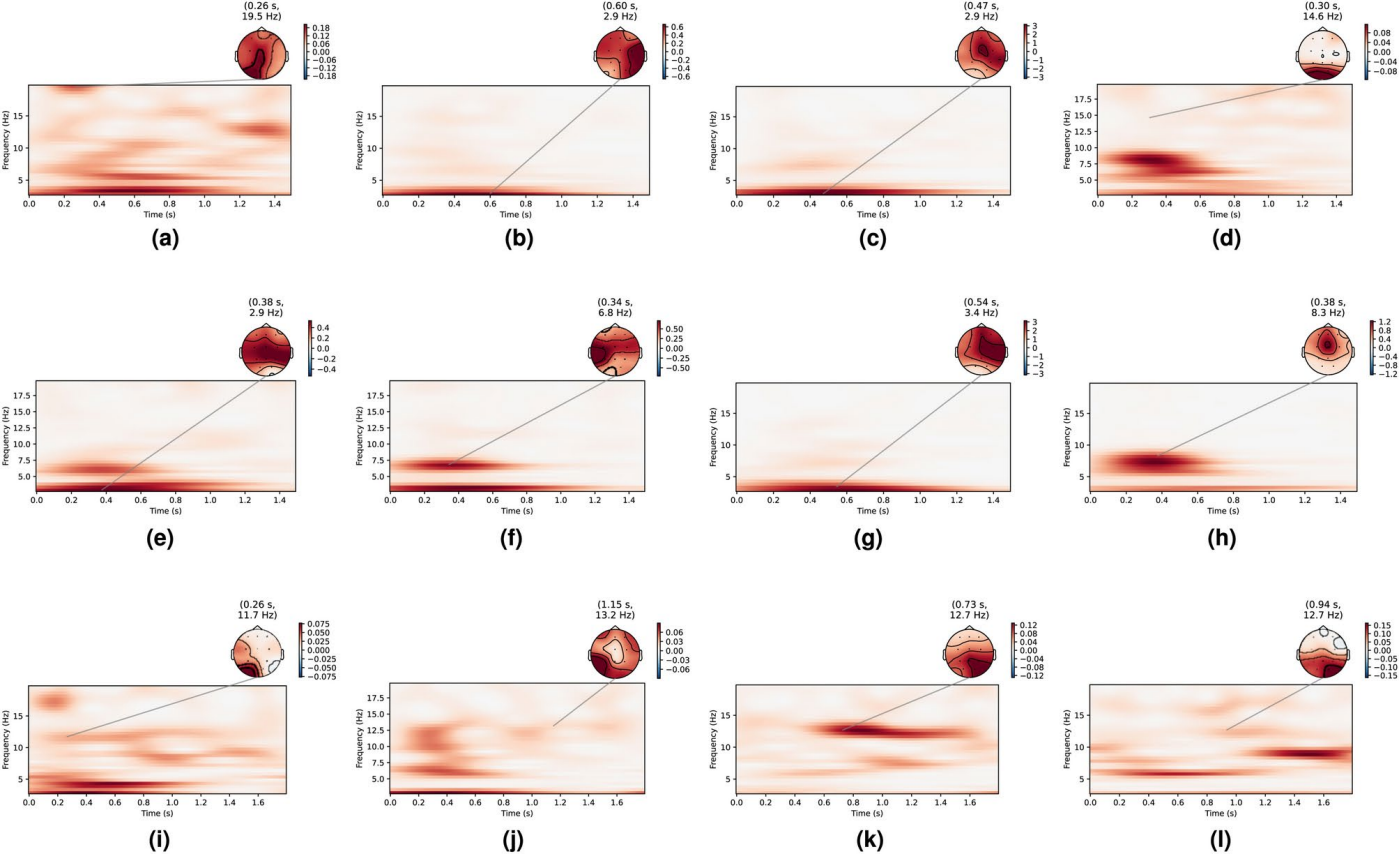
ErrP time-frequency plots for a few users in the errp3 dataset (**a**–**c**) and in the errp3 ssvep 14 dataset (**d**–**f**).

The time-frequency curves for these datasets are shown in Fig. 14, where it is evident that the SSVEP response for D4 and D5 is largely absent, unlike Fig. 5, where the 12.5Hz component was visible in the dataset. This absence could be attributed either to destructive interference during the averaging of non-phase-locked SSVEP signals or to poor SSVEP elicitation in general. The accuracy results for these datasets, calculated using our four state-of-the-art detection models following the same methodology described earlier, are presented in Tables 4 and 5.

A notable observation is that D3 shows higher accuracy than the baseline 1.5-second ErrP dataset (marginally higher for xDAWN and SSVEPNet but notably higher for EEGNet as seen in Table 3, while EEG-Deformer results were mixed). This improvement can be explained by two factors. First, doubling the time window from 1.5 to 3 s reduces the subject’s time pressure, allowing more time to mentally register errors before the agent proceeds with the next action, thus improving accuracy. Second, the increased consequence of each error may play a role. Since each error required the agent to correct its move, the duration of the episode was extended, forcing subjects to spend more time on the experiment, which may have increased the perceived consequence of an error, thereby enhancing the error response and by extension, the accuracy. In D5 (refer Table 5), where we used peripheral LED strip flickering at 14 Hz, the accuracy was similar to D3, with no significant differences observed across the 9 spatio-spectral permutations. While D5 had improved accuracy for all electrodes and 1-40Hz bandpass filtering condition, the improvement was not as pronounced in the remaining permutations. In contrast, D4, which used screen flickering at 7 Hz, exhibited noticeably lower accuracy compared to both D3 and D5 for all the spatio-spectral permutations (refer Table 4).

**Table 3:** Mean balanced accuracy performance for D1 (ErrP dataset with 1.5s action window) and D3 (ErrP dataset with 3s action window). Each dataset is evaluated along 9 permutations of electrodes (E1, E2, E3) and band-pass filtering (F1, F2, F3).

Dataset	Model	E1F1	E1F2	E1F3	E2F1	E2F2	E2F3	E3F1	E3F2	E3F3
D1	xDAWN + RG	71.17 ± 0.25	56.93 ± 0.38	57.21 ± 0.24	71.32 ± 0.18	57.14 ± 0.53	56.79 ± 0.44	66.90 ± 0.24	**55.41 ± 0.33**	**54.98 ± 0.39**
D3	xDAWN + RG	**73.34 ± 0.25**	**57.99 ± 0.34**	**57.45 ± 0.60**	**73.19 ± 0.28**	**58.22 ± 0.24**	**57.00 ± 0.34**	**67.60 ± 0.33**	**55.41 ± 0.44**	54.08 ± 0.47
D1	EEGNet	72.88 ± 0.51	68.55 ± 1.03	59.72 ± 0.80	70.80 ± 0.47	65.53 ± 0.56	56.94 ± 0.64	67.98 ± 0.46	**62.52 ± 0.39**	54.84 ± 0.82
D3	EEGNet	**77.82 ± 0.90**	**73.60 ± 1.44**	**62.38 ± 1.21**	**73.16 ± 0.91**	**72.14 ± 0.86**	**60.76 ± 0.41**	**70.53 ± 1.01**	60.37 ± 0.91	**57.84 ± 0.75**
D1	EEG-Deformer	**64.11 ± 1.89**	**54.5 ± 0.96**	54.5 ± 0.67	**61.71 ± 1.02**	**53.07 ± 0.75**	51.35 ± 0.36	61.01 ± 0.77	**54.46 ± 0.64**	53.12 ± 0.66
D3	EEG-Deformer	61.91 ± 1.36	51.32 ± 0.63	**54.6 ± 0.7**	58.07 ± 1.49	50.02 ± 0.17	**51.49 ± 0.54**	**63.3 ± 1.34**	53.26 ± 0.83	**54.9 ± 0.93**
D1	SSVEPNet	70.12 ± 0.68	56.32 ± 0.58	55.32 ± 0.78	61.34 ± 1.2	**52.62 ± 0.96**	52.42 ± 0.71	63.69 ± 1.01	55.21 ± 0.82	52.82 ± 0.44
D3	SSVEPNet	**71.07 ± 1.23**	**56.99 ± 0.77**	**56.94 ± 0.66**	**63.51 ± 1.05**	52.41 ± 0.48	**52.68 ± 0.54**	**70.43 ± 1.25**	**58.42 ± 1.05**	**56.45 ± 0.65**

Greater values are in bold.

**Table 4:** Mean balanced accuracy performance for D3 (ErrP dataset with 3s action window) and D4 (ErrP dataset with 3s action window and a 7Hz screen flickering stimulus) datasets with different flickering methods, flickering frequency, and ErrP action window. Each dataset is evaluated along 9 permutations of electrodes (E1, E2, E3) and band-pass filtering (F1, F2, F3) configurations.

Dataset	Model	E1F1	E1F2	E1F3	E2F1	E2F2	E2F3	E3F1	E3F2	E3F3
D3	xDAWN + RG	**73.34 ± 0.25**	**57.99 ± 0.34**	**57.45 ± 0.60**	**73.19 ± 0.28**	**58.22 ± 0.24**	**57.00 ± 0.34**	**67.60 ± 0.33**	**55.41 ± 0.44**	**54.08 ± 0.47**
D4	xDAWN + RG	63.07 ± 0.30	52.21 ± 0.55	53.04 ± 0.43	63.25 ± 0.26	52.44 ± 0.52	53.08 ± 0.38	57.78 ± 0.42	51.72 ± 0.35	51.94 ± 0.27
D3	EEGNet	**77.82 ± 0.90**	**73.60 ± 1.44**	**62.38 ± 1.21**	**73.16 ± 0.91**	**72.14 ± 0.86**	**60.76 ± 0.41**	**70.53 ± 1.01**	**60.37 ± 0.91**	**57.84 ± 0.75**
D4	EEGNet	67.49 ± 0.93	59.23 ± 1.05	55.01 ± 1.32	69.42 ± 0.65	62.55 ± 1.10	54.96 ± 1.08	60.24 ± 0.73	53.35 ± 1.67	53.85 ± 0.79
D3	EEG-Deformer	**61.91 ± 1.36**	**51.32 ± 0.63**	**54.4 ± 0.7**	**58.07 ± 1.49**	50.02 ± 0.17	**51.49 ± 0.54**	**63.3 ± 1.34**	**53.26 ± 0.83**	**54.9 ± 0.93**
D4	EEG-Deformer	55.67 ± 0.47	50.2 ± 0.44	52.79 ± 0.6	53.55 ± 0.81	**50.13 ± 0.46**	51.36 ± 0.44	55.57 ± 0.95	50.07 ± 0.4	52.39 ± 0.41
D3	SSVEPNet	**71.07 ± 1.23**	**56.99 ± 0.77**	**56.94 ± 0.66**	**63.51 ± 1.05**	**52.41 ± 0.48**	**52.68 ± 0.54**	**70.43 ± 1.25**	**58.42 ± 1.05**	**56.45 ± 0.65**
D4	SSVEPNet	59.85 ± 0.9	52.72 ± 1.01	52.72 ± 0.72	56.67 ± 0.55	51.33 ± 0.92	51.15 ± 0.43	59.36 ± 1.21	52.44 ± 0.7	52.44 ± 0.95

Greater values are in bold.

**Table 5:** Mean balanced accuracy performance for D3 (ErrP dataset with 3s action window) and D5 (ErrP dataset with 3s action window and a 14Hz LED flickering stimulus) datasets with different flickering methods, flickering frequency, and ErrP action window. Each dataset is evaluated along 9 permutations of electrodes (E1, E2, E3) and band-pass filtering (F1, F2, F3) configurations.

Dataset	Model	E1F1	E1F2	E1F3	E2F1	E2F2	E2F3	E3F1	E3F2	E3F3
D3	xDAWN + RG	73.34 ± 0.25	57.99 ± 0.34	**57.45 ± 0.60**	73.19 ± 0.28	**58.22 ± 0.24**	**57.00 ± 0.34**	67.60 ± 0.33	**55.41 ± 0.44**	**54.08 ± 0.47**
D5	xDAWN + RG	**75.06 ± 0.18**	**58.10 ± 0.45**	56.85 ± 0.27	**75.10 ± 0.21**	57.98 ± 0.31	56.72 ± 0.31	**67.92 ± 0.26**	53.81 ± 0.61	53.27 ± 0.43
D3	EEGNet	77.82 ± 0.90	73.60 ± 1.44	**62.38 ± 1.21**	73.16 ± 0.91	**72.14 ± 0.86**	**60.76 ± 0.41**	**70.53 ± 1.01**	60.37 ± 0.91	**57.84 ± 0.75**
D5	EEGNet	**80.83 ± 0.76**	**75.92 ± 0.68**	59.96 ± 0.99	**76.14 ± 0.80**	70.64 ± 1.04	60.60 ± 0.92	69.79 ± 1.11	**61.63 ± 1.73**	54.82 ± 1.45
D3	EEG-Deformer	61.91 ± 1.36	**51.32 ± 0.63**	**54.4 ± 0.7**	**58.07 ± 1.49**	50.02 ± 0.17	**51.49 ± 0.54**	**63.3 ± 1.34**	**53.26 ± 0.83**	**54.9 ± 0.93**
D5	EEG-Deformer	**63.42 ± 0.79**	50.98 ± 0.45	52.81 ± 0.29	56.72 ± 0.96	**51.97 ± 0.9**	51.32 ± 0.53	62.18 ± 1.18	52.51 ± 0.66	53.54 ± 0.83
D3	SSVEPNet	71.07 ± 1.23	**56.99 ± 0.77**	**56.94 ± 0.66**	**63.51 ± 1.05**	52.41 ± 0.48	**52.68 ± 0.54**	**70.43 ± 1.25**	**58.42 ± 1.05**	**56.45 ± 0.65**
D5	SSVEPNet	**71.15 ± 1.18**	55.13 ± 1.15	55.36 ± 0.74	63.24 ± 0.95	**53.24 ± 0.81**	52.42 ± 0.73	69.58 ± 0.97	55.36 ± 1.0	55.05 ± 0.72

Greater values are in bold.

While these experiments provided valuable insights, the vast number of possible parameter combinations limited our ability to explore all configurations. Future work will need to investigate how the method performs across a broader range of experimental and design parameters. To ensure consistency between the experimental and control datasets, we controlled for certain key factors, including experiment duration, participant age range, and an approximate gender balance within the group. However, several subject-related factors were not controlled. Specifically, prior exposure to the experimental protocol and familiarity with video games were not included as control variables, potentially affecting participant performance. Additionally, while we approximately controlled for age range and gender ratio, factors such as handedness were not standardized across participants. Psychological parameters-such as fatigue, mood, motivation, and distraction levels-along with individual cognitive baselines (we did not control for it aside from the fact that all the participants that were recruited were graduate students enrolled in the same university) and psychological traits were also unaccounted for. Finally, we did not control for past experience with BCI devices or for the time of day during which experiments were conducted. These uncontrolled factors may introduce variability in participant responses across sessions. Additionally, there are several important areas for further research. First, the potential to interface ErrP with other signals, such as P300, remains unexplored. Optimization of flicker frequencies and elicitation methods is also needed to determine the best coupling between two signals. Although SSVEP signals typically have a phase-locked response with their stimulus, the impact of artifacts such as eye blinks on SSVEP fluctuations requires deeper investigation. Additionally, further research can also explore the optimum duty cycle for SSVEP flickering that provides an improvement in performance without fatiguing a user significantly. There is also significant potential to explore how SSVEPs might synchronize EEG responses in more naturalistic settings. Regarding data quality control, while we demonstrated how fidelity scores could inform user data quality, further research is needed to assess whether SSVEP-based fidelity scores can evaluate dataset quality at a more granular level, potentially on a per-trial or per-episode basis. This would allow the rejection of only noisy samples while retaining higher-quality data. Additionally, while we use the SSVEP-based fidelity score to gauge data quality in a broad sense, we have yet not studied the impact of artifacts like eye movements and muscle movements on the quality of this metric. We think it is a very important avenue as for instance, eye-blinks can impact this metric in two ways. Firstly, by adding an artifact and therefore an interference in the EEG signal, and secondly, by interfering with the flashing sequence that is visible to the human eye. Among other questions, it is worth exploring how blinking impairs the SSVEP magnitude in the occipital lobes due to the user closing their eyes and thus, being unreceptive to the flashing stimulus. The potential impact of eye-blinks and other eye and muscle-related artifacts on this metric opens a very pertinent avenue for research questions to be answered that we leave it to parallel research endeavors and future work. Additionally, we recognize that a more detailed exploration of the interaction between SSVEPs and ErrPs is crucial. One particularly important dimension is the quantification of visual fatigue, which may arise from prolonged exposure to flickering stimuli. Such fatigue could have detrimental effects on ErrP elicitation, reducing the strength and reliability of the responses over time. Another factor is the potential for distraction, where subjects may preferentially allocate their attention to the visually salient SSVEP stimulus, thereby reducing focus on the movement-based ErrP stimulus. This could lead to inhibited ErrP elicitation, particularly when both stimuli are presented concurrently. Understanding these factors is essential for designing more effective concurrent stimuli that optimize the performance of both SSVEP and ErrP systems. Further research into these dimensions will contribute to a more comprehensive understanding of the interplay between SSVEPs and ErrPs, ultimately enhancing BCI system efficiency.

## Supplementary Information

Supplementary Information.

## Data Availability

We make two datasets available for this manuscript. The first dataset is fully open-access and can be accessed at IEEE Dataport (10.21227/7rwy-9q83) The second dataset is provided within the manuscript or supplementary information files.
