# Supplementary material for: Response coupling with an auxiliary neural signal for enhancing brain signal detection
Source: Sci Rep. 2025 Feb 20;15:6227. doi: 10.1038/s41598-025-87414-9 (PMC11842634; doi:10.1038/s41598-025-87414-9)
Supplement: Supplementary file 1 — Supplementary Information. [file 41598_2025_87414_MOESM1_ESM.pdf]

Supplementary Information: Response coupling

Ekansh Gupta and Raghupathy Sivakumar

Georgia Institute of Technology, Atlanta, Georgia, USA

Synchronization

System equations for cross-correlation maxima indices

Experimentally, the aggregate SSVEP signals exhibit a PLV ranging from 0.85 to 0.99 across users, as mentioned in the main article. An example value of sliding PLV for a time duration of 0.5 seconds (62 samples) across the larger time window of 1.8 seconds in shown in Fig 1b. This high PLV signifies that the average response of an electrode is phase-locked with an almost constant phase lag with respect to the stimulus signal. We now put forward our system model for calculating phase lag and response lag using a cross-correlation between the response and stimulus. As our synthetic stimulus also contains non-ssvep zones, it can be represented as the following (using a step function that goes to zero when the flickering is not active).

$$S(t) = A_0 * sin(2\pi F_0 t) * \text{Step}(t), \quad t \in [0, T_0] \tag{1}$$

$$\text{Where Step}(t) = \begin{cases} 1, & \text{if } t <= T_S \\ 0, & \text{otherwise} \end{cases}, \quad t \in [0, T_0] \tag{2}$$

For N repetitions of the stimulus, this function repeats with a period of T_0. At any given instant, the EEG response of an electrode can simply be denoted by

$$R(t) = R_0(t) + N(t), \quad t \geq 0 \tag{3}$$

Where, $R_0(t)$ is the signal portion of the response and $N(t)$ is the noise component. The cross-correlation value of the SSVEP response with the synthetic stimulus signal repeated for N cycles can then be denoted as:

$$C(\tau) = \int_{\tau}^{NT_0+\tau} A_0 * sin(2\pi F_0(t-\tau)) * \text{Step}(t-\tau) * [R_0(t) + N(t)]\, dt \tag{4}$$

This can be broken down into N periods of time duration T_0 and can be rearranged as the following:

$$\begin{aligned} C(\tau) = &\int_{\tau}^{T_0+\tau} A_0 * sin(2\pi F_0(t-\tau)) * \text{Step}(t-\tau) * [R_0(t) + N(t)]\, dt + \\ &\int_{T_0+\tau}^{2T_0+\tau} A_0 * sin(2\pi F_0(t-\tau)) * \text{Step}(t-\tau) * [R_0(t) + N(t)]\, dt + \\ &\cdots\cdots\cdots + \\ &\int_{(N-1)T_0+\tau}^{NT_0+\tau} A_0 * sin(2\pi F_0(t-\tau)) * \text{Step}(t-\tau) * [R_0(t) + N(t)]\, dt \end{aligned} \tag{5}$$

$$C(\tau) = \int_{\tau}^{T_0+\tau} \Sigma_{i=0}^{N-1} A_0 * sin(2\pi F_0(t + iT_0 - \tau)) * \text{Step}(t + iT_0 - \tau) * [R_0(t + iT_0) + N(t + iT_0)] dt \tag{6}$$

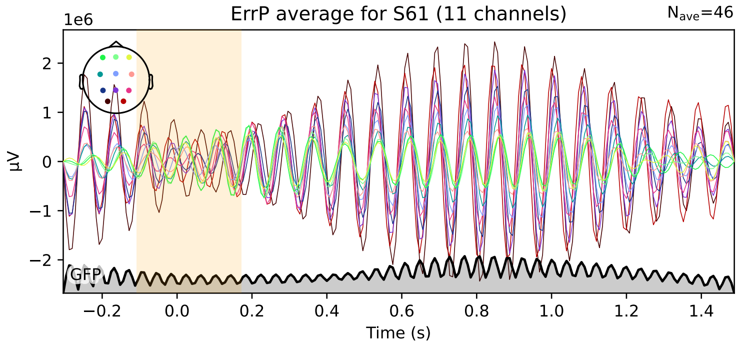

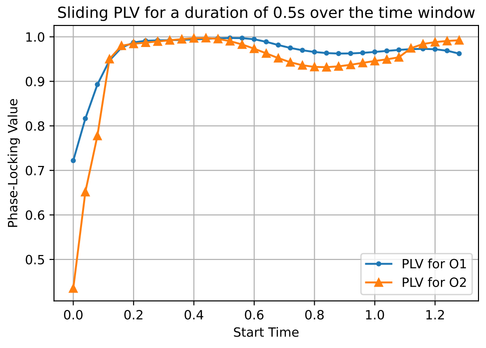

(a) Averaged ErrP signal with no-SSVEP zone highlighted **(b)** PLV as a function of time for the averaged signal

Figure 1. Average ErrP response signals bandpass filtered from 11-14Hz for a typical subject with the 200ms non-SSVEP zone highlighted in yellow as well as the PLV as a function of time

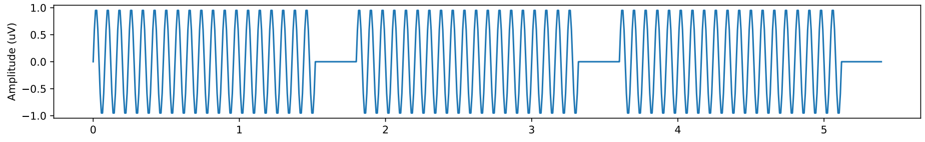

(a) Synthetic stimulus signal with 3 repetition cycles

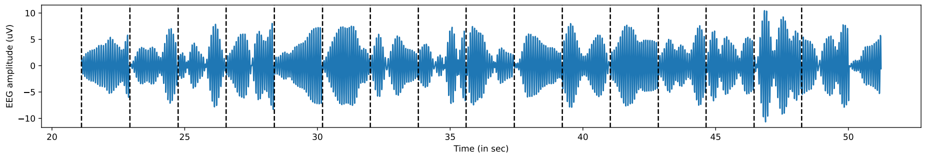

(b) An example of band-pass filtered EEG for O1 and O2 electrodes

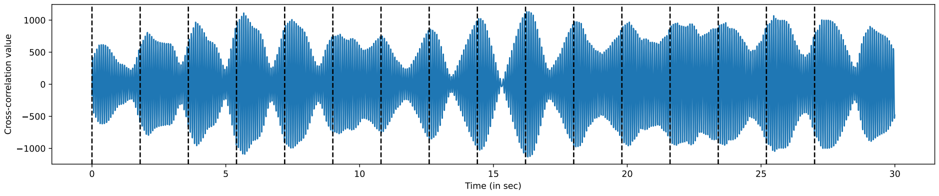

(c) Cross-correlation result between synthetic stimulus signal and bandpass filtered EEG for the O1 electrode

Figure 2. Stimulus signal cross-correlated with bandpass filtered O1 EEG

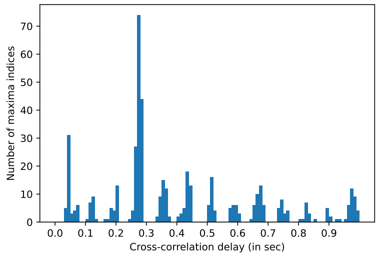

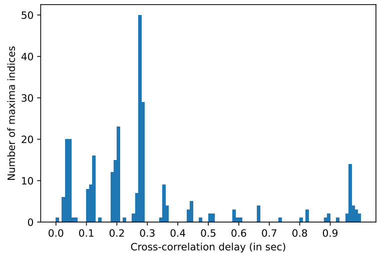

(a) Histogram of indices of cross-correlation maxima **(b)** Histogram of indices of cross-correlation maxima

Figure 3. Histograms of indices of cross-correlation maxima for 2 users

Note that the synthetic stimulus term in the summation, $A_0 * sin(2\pi F_0(t+iT_0-\tau)) * \text{Step}(t+iT_0-\tau)$ is equal to $A_0 * sin(2\pi F_0(t-\tau)) * \text{Step}(t-\tau)$ as it repeats itself every T_0 as seen in Fig 2a. Additionally, we can denote $R_0(t+iT_0)$ by $R_i(t)$, $N(t+iT_0)$ by $N_i(t)$. The equation then simplifies to:

$$C(\tau) = \int_{\tau}^{T_0+\tau} \Sigma_{i=0}^{N-1} A_0 * sin(2\pi F_0(t-\tau)) * \text{Step}(t-\tau) * [R_i(t) + N_i(t)]\, dt \tag{7}$$

Rearranging the limits, this can be written as:

$$C(\tau) = \int_{0}^{T_0} \Sigma_{i=0}^{N-1} A_0 * sin(2\pi F_0 t) * \text{Step}(t) * [R_i(t+\tau) + N_i(t+\tau)]\, dt \tag{8}$$

$$C(\tau) = \int_{0}^{T_0} A_0 * sin(2\pi F_0 t) * \text{Step}(t) * \Sigma_{i=0}^{N-1} [R_i(t+\tau) + N_i(t+\tau)]\, dt \tag{9}$$

The term inside the summation, $\Sigma_{i=0}^{N-1} R_i(t+\tau) + N_i(t+\tau)$ represents the sum of N trials of signals from τ to $T_0+\tau$. This is equal to N times the mean signal obtained after taking the average of N trials. This response looks like the one shown in Fig 1a. Also, this signal exhibits a high phase locking value ranging from 0.85 to 0.99 which means the phase lag throughout the time window is relatively constant. Because all dipole sources are assumed to oscillate in phase, their individual phase lags are a function of their individual propagation delays[1]. The aggregate response of multiple (K) cortical sources can then be modeled as the linear superimposition of the contributions from the individual dipoles:

$$\bar{R(t)} = \Sigma_{i=1}^{K} A_i(t) * S_0(t-\tau_i) = \Sigma_{i=1}^{K} A_i(t) * sin(2\pi F_0(t-\tau_i)) \tag{10}$$

Since a linear combination of sinusoids of a fixed frequency can be represented as a singular sinusoidal component, this can further be broken down and represented as the following with a certain amplitude and phase lag. Note that $\bar{\Phi}$ is treated like be constant (due to the phase-locking value) and thus not written as a function of time.

$$\bar{R(t)} = \bar{A}(t) * sin(2\pi F_0 t + \bar{\Phi}) + N(t) \tag{11}$$

In light of this, the summed response can be written as:

$$C(\tau) = N * \int_{0}^{T_0} A_0 * sin(2\pi F_0 t) * \text{Step}(t) * \left[\bar{A}(t+\tau) * sin(2\pi F_0(t+\tau) + \bar{\Phi}) + \bar{N}(t+\tau)\right] dt \tag{12}$$

We can expand the Step(t) function and this results in the integral running from 0 to T_S.

$$C(\tau) = NA_0 * \int_{0}^{T_S} sin(2\pi F_0 t) * \left[\bar{A}(t+\tau) * sin(2\pi F_0(t+\tau) + \bar{\Phi}) + \bar{N}(t+\tau)\right] dt \tag{13}$$

$$\begin{aligned} C(\tau) = NA_0 * \int_{0}^{T_S} \bar{A}(t+\tau) * \left[sin^2(2\pi F_0 t) * cos(2\pi F_0 \tau + \bar{\Phi}) + sin(2\pi F_0 t) * cos(2\pi F_0 t) * sin(2\pi F_0 \tau + \bar{\Phi})\right] \\ + sin(2\pi F_0 t) * \bar{N}(t+\tau) dt \end{aligned} \tag{14}$$

$$\tau = (-\delta + 0.08n), \quad n = 1, 2, 3, .. \tag{15}$$

Assuming the noise component is small (after averaging N trials), we can simplify the expression to the following:

$$\begin{aligned} C(\tau) = N * A_0 * cos(2\pi F_0 \tau + \bar{\Phi}) * \left[\int_{0}^{T_S} \bar{A}(t+\tau) * sin^2(2\pi F_0 t) dt\right] + N * A_0 * sin(2\pi F_0 \tau + \bar{\Phi}) * \\ \left[\int_{0}^{T_S} \bar{A}(t+\tau) * sin(2\pi F_0 t) * cos(2\pi F_0 t) dt\right] \end{aligned} \tag{16}$$

$$C(\tau) = N * A_0 * cos(2\pi F_0 \tau + \bar{\Phi}) * \left[\int_0^{T_S} \bar{A}(t+\tau) * sin^2(2\pi F_0 t) dt \right] + N * A_0 * sin(2\pi F_0 \tau + \bar{\Phi}) * \left[\int_0^{T_S} \bar{A}(t+\tau) * \frac{sin(4\pi F_0 t)}{2} dt \right] \tag{17}$$

For the second term in the integral, $\int_0^{T_S} \bar{A}(t+\tau) * sin(4\pi F_0 t) dt$, the sine term varies with the periodicity of $2F_0$, i.e. twice as fast as the SSVEP frequency. In one full period of the sine term, i.e. in half a cycle of the SSVEP signal, the envelope term $\bar{A}(t+\tau)$ remains relatively constant compared to the sinusoid term. Refer to Fig 1a to see that in one cycle of the SSVEP signal, the envelope term is significantly slow-changing compared to the sinusoid variation dependent on F_0. Thus, this can be viewed as a piecewise integral of $sin(4\pi F_0 t)$ across multiple periods of length $1/4F_0$. Since the integral of $sin(\theta)$ vanishes in one period, this integral vanishes overall and contributes little to the cross-correlation expression. Thus, we are left with the following term.

$$C(\tau) = N * A_0 * cos(2\pi F_0 \tau + \bar{\Phi}) * \left[\int_0^{T_S} \bar{A}(t+\tau) * sin^2(2\pi F_0 t) dt \right] \tag{18}$$

References

1. Srinivasan, R., Bibi, F. A. & Nunez, P. L. Steady-state visual evoked potentials: distributed local sources and wave-like dynamics are sensitive to flicker frequency. *Brain Topogr.* **18**, 167–187, DOI: 10.1007/s10548-006-0267-4 (2006).
